# ZCCHC4 Orchestrates Hepatocellular Carcinoma Metastasis by Regulating Lipid biosynthesis and TMEM97/LCN2/Twist1 Pathway

**DOI:** 10.7150/ijbs.120086

**Published:** 2026-03-25

**Authors:** Juan Ye, Xinyi Wen, Ruiyang Liu, Caini Huang, Zhijie Xu, Yi Zhan, Fuyuan Xu, Hongbin Huang, Chunhui Qi, Yao Tang, Peirong Li, Jianzhong He, Zhiju Zhao, Gang Lu, Fei Xiao

**Affiliations:** 1 Department of Infectious Diseases, The Fifth Affiliated Hospital, Sun Yat-sen University, Zhuhai, Guangdong Province, China.; 2 Guangdong Provincial Engineering Research Center of Molecular Imaging, The Fifth Affiliated Hospital, Sun Yat-sen University, Zhuhai, Guangdong Province, China.; 3 Guangdong-Hong Kong-Macao University Joint of Interventional Medicine, The Fifth Affiliated Hospital, Sun Yat-sen University, Zhuhai, Guangdong Province, China.; 4 Department of Pathology, The Fifth Affiliated Hospital, Sun Yat-sen University, Zhuhai, Guangdong Province, China.; 5 Key Laboratory for Regenerative Medicine, Ministry of Education, School of Biomedical Sciences, Faculty of Medicine, The Chinese University of Hong Kong, Hong Kong SAR, China.; 6 Kashi Guangdong Institute of Science and Technology, The First People's Hospital of Kashi, Kashi, Xinjiang Uyghur Autonomous Region, China.; 7 State Key Laboratory of Anti-Infective Drug Development, School of Pharmaceutical Sciences, Sun Yat-sen University, Guangzhou, Guangdong Province, China.; 8 Department of Oncology, The Second Affiliated Hospital of Zunyi Medical University, Zunyi, Guizhou Province, China.

**Keywords:** Hepatocellular carcinoma metastasis, Lipid metabolism, Wnt pathway, EMT, ZCCHC4, TMEM97.

## Abstract

Metastasis remains a major therapeutic challenge in hepatocellular carcinoma (HCC), yet its underlying molecular mechanisms are not fully understood. This study reveals that ZCCHC4 acts as a key promoter of HCC metastasis. We demonstrate that ZCCHC4 enhances the metastatic capacity of HCC cells both *in vitro* and *in vivo*. Mechanistically, ZCCHC4 upregulates key lipid biosynthesis enzymes (HMGCR, SQLE, FASN, and SCD), leading to intracellular accumulation of cholesterol and fatty acids which drive metastasis. Concurrently, ZCCHC4 transcriptionally activates TMEM97, thereby augmenting Wnt signaling. Furthermore, TMEM97 interacts with LCN2 to retain it in the cytoplasm. This sequestration relieves LCN2-mediated inhibition of the transcription factor Twist1, consequently promoting epithelial-mesenchymal transition (EMT) and metastasis. Consistent with these findings, ZCCHC4 level positively correlates with levels of TMEM97 and the lipid enzymes in clinical HCC specimens, and high ZCCHC4 expression is associated with poor patient prognosis. In summary, our work identifies ZCCHC4 as a critical metastasis driver via coordinated regulation of lipid metabolism and the TMEM97/LCN2/Twist1 axis, presenting novel potential targets for treating HCC metastasis.

## Introduction

Primary liver cancer, one of the most common malignancies worldwide, is projected to account for approximately 866,000 new cases and 759,000 deaths by 2025, ranking eighth in incidence and fourth in mortality among all cancers 1. Hepatocellular carcinoma (HCC) constitutes over 80% of primary liver cancer cases 2. Although HCC typically presents as a liver-confined lesion, distant metastasis is frequently detected-up to 68% of patients show metastatic disease at autopsy, and many develop metastases during or after treatment 3. Despite available therapies, the overall mortality rate for metastatic HCC remains as high as 90% 2. Therefore, understanding the mechanisms driving HCC metastasis and identifying novel therapeutic targets are urgently needed.

Metastasis is a multi-step process known as the invasion metastasis cascade 4. Metabolic reprogramming-encompassing glucose, nucleic acid, and lipid metabolism-plays a key role in this process 5. In particular, lipid metabolic reprogramming has emerged as a hallmark of cancer, involving altered lipid uptake, synthesis, breakdown, and storage. These changes support energy production, membrane remodeling, and signaling, enabling tumor cells to acquire invasive traits and modify the tumor microenvironment to facilitate metastasis 6. In HCC, lipid reprogramming not only remodels the tumor microenvironment but also disrupts cellular lipid homeostasis, leading to aberrant activation of pro-metastatic signaling pathways 7, 8. In addition, Epithelial mesenchymal transition (EMT) is another critical driver of metastasis 7. EMT is regulated by a complex network of transcription factors, epigenetic modifiers, non-coding RNAs, and signaling pathways such as Wnt, TGF-β, and NOTCH 9. Upon EMT induction, epithelial cells undergo cytoskeletal reorganization and morphological changes, resulting in loss of cell-cell adhesion and enhanced motility, thereby promoting invasion and dissemination 10. EMT is modulated by EMT-inducing transcription factors (EMT-TFs) such as SNAIL, Twist, ZEB, and Slug, which promote the expression of markers associated with mesenchymal cells, such as vimentin and N-cadherin, while inhibiting the expression of markers associated with epithelial cells, such as E-cadherin and Claudin-1, promote metastasis 11. Furthermore, the Wnt/β-catenin pathway plays a central role in regulating EMT 12, 13. In HCC, multiple regulators converge on this axis: UBE2T promotes EMT and metastasis via the MAPK-ERK/β-catenin pathway 14. PSPC1 upregulation or PSPC1-Y523F mutation drives cytoplasmic translocation of PTK6 and nuclear accumulation of β-catenin 15. NCSTN, TXNDC12, and GINS1 each facilitate β-catenin nuclear translocation, activating ZEB1-mediated EMT and metastasis 16-18. Additionally, lncRNA DLGAP1-AS modulates miR-26a/b-5p and its targets CDK8 and LRP6 to activate Wnt/β-catenin signaling and promote EMT-driven metastasis 19. In summary, targeting key molecules involved in lipid metabolism, EMT, and Wnt signaling represents a promising strategic approach for treating HCC metastasis.

Zinc-finger proteins (ZFPs) are the largest family of transcription factors in human genome, which have a wide range of molecular functions due to their different zinc finger domains. In addition to binding to DNA, ZFPs can also interact with RNA, PAR (poly adp-ribose), proteins and lipids, mediate transcription or post-transcriptional regulation, and participate in the regulation of tumor occurrence and development 20. ZFPs have been increasingly implicated in metabolic reprogramming, epithelial-mesenchymal transition (EMT), metastasis, immunity, cell cycle, apoptosis, and stemness 21. ZCCHC4 (Zinc Finger CCHC-Type Containing 4) is a typical ZFP member. It contains a conserved catalytic DPPF domain, which is predicted to confer N6-methyladenosine (m^6^A) methyltransferase activity, as well as a CCHC-Znf domain and a Zf-GRF domain 22, 23. As a zinc finger protein, ZCCHC4 has been reported to regulate proliferation, apoptosis, and chemosensitivity in several cancers 22, 24, 25. However, its role and mechanism in cancer metastasis remain poorly understood. In HCC, previous studies indicated that ZCCHC4 promotes tumor cell growth 22 and enhances chemoresistance by disrupting the interaction between lncRNA AL133467.2 and γH2AX, thereby inhibiting DNA damage-induced apoptosis 24. Nevertheless, how ZCCHC4 influences HCC metastasis has not been characterized.

In this study, we found that ZCCHC4 is significantly upregulated in HCC tissues, and its high expression correlates with poor clinical outcomes. Functional experiments demonstrated that ZCCHC4 promotes EMT and metastatic dissemination in HCC. Mechanistically, ZCCHC4 transcriptionally activates key genes involved in cholesterol and fatty acid biosynthesis and upregulates the Wnt pathway activator TMEM97, collectively driving EMT and metastasis. Moreover, we identified that TMEM97 interacts with LCN2 to sequester LCN2 in the cytoplasm, thereby relieving its suppression of the EMT-related transcription factor Twist1. These results reveal that ZCCHC4 orchestrates HCC metastasis through multiple signaling axes and highlights its potential as a therapeutic target for suppressing metastatic HCC.

## Materials and Methods

### Human clinical specimens

Primary hepatocellular carcinoma (HCC) tissues and their corresponding adjacent non-cancerous liver tissues were obtained from patients who underwent surgical resection at the Fifth Affiliated Hospital of Sun Yat-sen University. Paraffin-embedded sections and relevant clinical data were procured from the Department of Pathology and the Medical Records Department of the same institution, respectively. The inclusion criteria for study participants were defined as follows: (A) Histopathologically confirmed HCC diagnosis by the institutional pathology department, without evidence of other concurrent malignancies; (B) No prior anti-tumor therapy administered before surgical intervention; (C) Preoperative evaluations confirming the absence of surgical contraindications; (D) Availability of complete clinical data and follow-up records. Patients were excluded if they met any of the following criteria: A. Diagnosis of additional malignancies alongside confirmed HCC. B. Presence of severe comorbid systemic diseases. C. Incomplete clinical records or follow-up information. All human specimen collection procedures were conducted in accordance with the ethical guidelines outlined in the Declaration of Helsinki. The procurement and utilization of all human specimens were sanctioned by the Committee for Ethical Review of Research at the Fifth Affiliated Hospital of Sun Yat-sen University (No.2022-K105-1).

### Targeted metabolomics methods

HCC cells were harvested and immediately snap-frozen for storage at -80 °C until subsequent analysis. Targeted metabolomics analysis was conducted by Shanghai Applied Protein Technology (Shanghai, China) using an AB 6500+ QTRAP triple quadrupole-linear ion trap mass spectrometer (AB SCIEX, Framingham, MA, USA). Raw mass spectrometric data were acquired and processed using Sciex OS analytical software (AB SCIEX).

### CUT&Tag and data analysis

CUT&Tag analysis was executed utilizing the Hyperactive In-Situ ChIP Library Prep Kit (cat. TD901-01, Vazyme) per the supplier's protocols. In summary, 100,000 cells underwent harvesting and washing with Wash buffer containing protease inhibitor cocktail. The cells were then bound to ConA magnetic beads, placed in antibody dilution buffer, and left to interact with Anti-Flag antibody (F1804, Sigma) overnight at 4 °C. After washing away excess primary antibodies, the ConA-bound cells were placed in Dig-wash buffer with secondary antibody and maintained at ambient temperature for 2 h. Post three Dig-wash buffer cleanses, the samples were transferred to Dig-300 buffer containing Hyperactive pG-Tn5/pA Transposon and kept at room temperature for 2 h. The cells then underwent Dig-300 buffer washing before being transferred to Tagmentation Buffer for 37 °C incubation lasting 1 h. The tagmentation process was stopped by introducing 10 µl of 0.5 M EDTA, 3 µl of 10% SDS, and 2.5 µl of 20 mg/ml Proteinase K, succeeded by 55 °C incubation for 1 h. DNA extraction proceeded via the phenol-chloroform technique. The library amplification employed PCR under these conditions: 72 °C for 3 min; 98 °C for 30 s; 15 cycles at 98 °C for 15 s, 60 °C for 30 s, and 72 °C for 30 s; concluding with 72 °C for 5 min. The libraries underwent purification utilizing 1.2× AMPure XP beads (cat. A63881, Beckman). Quality evaluation was completed using the Agilent 2200 system. Sequencing was executed on the Illumina NovaSeq 6000 platform.

CUT&Tag sequencing data were mapped to the hg38 human genome assembly through the short-read aligner Bowtie. Peak calling was conducted with MACS (version 2.1.1.20160309) under a statistical threshold of *p* < 0.001, and the resulting data were visualized using the Integrative Genomics Viewer (version 2.8.9, Broad Institute). Promoter regions were denoted as ±2 kb versus the transcription start site (TSS). Motif enrichment analysis was carried out using the HOMER software suite.

### ChIP-quantitative PCR (ChIP-qPCR)

ChIP analysis was executed utilizing the SimpleChIP Plus Enzymatic Chromatin IP Kit (#9005, CST) per the supplier's protocols. In brief, PLC/PRF/5 cells expressing Flag-tagged ZCCHC4 underwent crosslinking treatment with 1% formaldehyde during a 10-min period at ambient temperature, with subsequent glycine (0.125 M) quenching applied for 5 min. The processed cells received ice-cold phosphate-buffered saline (PBS) washing and underwent centrifugation at 300 × g for 5 min under 4 °C conditions. The obtained cell pellets underwent resuspension in Buffer A containing DTT plus protease inhibitors, followed by ice incubation for 10 min and centrifugation at 1800 × g for 5 min at 4 °C. The resulting pellet was resuspended in Buffer B containing DTT and digested with Micrococcus nuclease for 25 min at 37 °C to fragment chromatin to a size range of 150-900 bp. EDTA addition terminated the reaction. Nuclear pellets were then resuspended in ChIP Buffer C with protease inhibitors and subjected to sonication to disrupt the nuclear membrane. The resulting lysates were centrifuged at 9400 × g for 10 min at 4 °C, and the supernatant was procured. A 10 µl aliquot of the supernatant was stored at -20 °C for use as input DNA. Anti-Flag antibody (F1804, Sigma) and Anti-IgG antibody (A7028, Beyotime) were added separately to the remaining supernatant and incubated overnight at 4 °C with rotation. Subsequently, Protein G Magnetic Beads were introduced and underwent rotational incubation at 4 °C for 2 h. The precipitated complexes received sequential washing treatments using low-salt buffer, high-salt buffer, LiCl buffer, and Tris-EDTA buffer. The DNA components were extracted through ChIP Elution Buffer, followed by overnight reversal of crosslinks. Treatment with RNase A eliminated RNA content, while Proteinase K degraded protein components. The final DNA isolation utilized spin columns, after which qPCR analysis was performed. Three groups of DNA fragments—5% input, anti-IgG, and anti-Flag—were used as templates for enrichment comparison. qPCR was executed utilizing TB Green Premix Ex Taq II (RR820A, TAKARA, Japan) on the QuantStudio 7 Flex Real-Time PCR System (Thermo). The amplification conditions were 95 °C for 30 s, succeeded by 40 cycles of 95 °C for 5 s and 60 °C for 30 s. The anti-IgG group was included as a negative control. Primer sequences targeting specific promoter regions are depicted in Table S5.

### Animal experiments

Male BALB/c Nude mice (4-6 weeks old) were obtained from Vital River Laboratory Animal Technology Co., Ltd. (Beijing, China). All animal-related procedures were approved by the Institutional Animal Care and Use Committee and conducted at the Guangdong Provincial Key Laboratory of Biomedical Imaging, Fifth Affiliated Hospital of Sun Yat-sen University, per institutional ethical guidelines (approval No. 00117).

A total of 1 × 10⁶ stably transfected cells were injected via the tail vein. After 10 weeks, the mice were sacrificed, and their lungs were harvested. The lung specimens underwent photography, fixation using 4% paraformaldehyde, embedding in paraffin, sectioning procedures, and subsequent hematoxylin and eosin staining. The number of metastatic lung nodules was quantified following microscopic imaging.

A total of 5 × 10⁶ stably transfected cells were subcutaneously injected into the left axillary region of the mice. Tumor volumes were determined utilizing the formula: V (mm³) = (width² (mm²) × length (mm)) / 2. After 4 weeks, the mice were euthanized, and the tumors were excised for subsequent analyses.

Male BALB/c-nude mice (3-5 weeks old) were used to establish an orthotopic transplantation model of HCC. HCC cells (1 × 10⁶ cells per mouse) with stable shScramble or shZCCHC4 transfection were resuspended in Matrigel (Corning, Cat. No. 354277), and the resulting cell suspension was orthotopically implanted into the liver lobes of nude mice (n = 6 for the shScramble group and n = 6 for the shZCCHC4 group). To prevent leakage of tumor cells, gentle pressure was applied to the injection site for 2 min. Five weeks following implantation, all mice were euthanized. For bioluminescent imaging, BALB/c-nude mice were anesthetized at defined time points post-transplantation, and D-Luciferin potassium salt (0.15 mg/g; HY-12591B, MCE) was administered via intraperitoneal injection. Bioluminescence images were acquired 10 min after substrate administration using the IVIS Lumina Ⅲ imaging system per the supplier's protocol.

### Immunohistochemical staining

Paraffin-embedded tissue sections with a thickness of 4 μm were serially sliced and mounted onto silicified glass slides. Following dewaxing and hydration, endogenous peroxidase activity was quenched using 3% hydrogen peroxide. Antigen retrieval was subsequently performed by incubating the sections in antigen repair solution (C1034, Solarbio, China) for 15 min. Nonspecific binding sites were then blocked with goat serum (ZLI-9022, ZSGB-BIO, China) for 30 min. The tissue sections were incubated overnight at 4 °C with the following primary antibodies: anti-ZCCHC4 (HPA035576, Sigma, USA), anti-TMEM97 (26444-1-AP, Proteintech, China), anti-*HMGCR* (13533-1-AP, Proteintech, China), anti-SCD (A16429, ABclonal, China), anti-*FASN* (10624-2-AP, Proteintech, China), anti-SQLE (12544-1-AP, Proteintech, China), anti-E-Cadherin (3195S, CST, USA), anti-Claudin-1 (13995S, CST, USA) and anti-Vimentin (5741S, CST, USA) antibodies. After removal of unbound primary antibodies, incubation with secondary antibodies (MXB, KIT-5020, China) was carried out at room temperature for 30 min. DAB (ZSGB-BIO, ZLI-9017, China) was applied for chromogenic development, and hematoxylin was utilized for nuclear counterstaining. The sections were then dehydrated, cleared, sealed, and imaged under a microscope. Two independent pathologists scored the staining based on both intensity and the percentage of positively stained area. The staining intensity (X) was graded as follows: 0 = no signal, 1 = weak, 2 = moderate, 3 = strong. The positive staining area (Y) was scored as: 0, < 5%; 1, 6-25%; 2, 26-50%; 3, 51-75%; 4, > 75%. The final IHC score was calculated by multiplying X and Y.

### RNA isolation and RNA-seq

Total RNA was extracted through RNAzol® RT (RN190, MRC, USA) per the supplier's protocol. The extracted RNA was subsequently submitted to Novogene for RNA-seq and downstream bioinformatic analysis. Sequencing was carried out using the Illumina HiSeq platform.

### Western blot

Total protein was procured utilizing RIPA buffer (P0013B, Beyotime, China). Protein concentrations were ascertained with the BCA Protein Assay Kit (23227, Thermo, USA). An amount of 50 μg of protein per sample was loaded onto 4-20% SDS-PAGE gels for separation, followed by transfer onto PVDF membranes. The following primary antibodies were utilized: anti-ZCCHC4 (ab209901, Abcam, UK), anti-ZCCHC4 (ab211325, Abcam, UK), anti-TMEM97 (26444-1-AP, Proteintech, China), anti-LCN2 (ab63929, Abcam, UK), anti-HMGCR (A1633, abclonal, China), anti-SCD (A16429, ABclonal, China), anti-FASN (10624-2-AP, Proteintech, China), anti-SQLE (12544-1-AP, Proteintech, China), anti-GSK-3β (12456T, CST, USA), anti-pGSK-3β (5558T, CST, USA), anti-active β-catenin (8814S, CST, USA), anti-E-Cadherin (3195S, CST, USA), anti-Claudin-1 (13995S, CST, USA), anti-Vimentin (5741S, CST, USA), anti-Twist1 (25465-1-AP, Proteintech, China), anti-Lamin B1(12987-1-AP, Proteintech, China), anti-AXIN2 (20540-1-AP, Proteintech, China) and anti-GAPDH (ab181602, Abcam, UK). Protein bands were detected through visualization utilizing the GenSuper ECL Ultra Western HRP Substrate (JXE0011, Genesion, China).

### Co-immunoprecipitation (CoIP) and mass spectrometry analysis

Protein was procured utilizing NP-40 buffer (P0013F, Beyotime, China). Protein concentration was quantified utilizing the BCA Protein Assay Kit. Anti-TMEM97 (#62790, CST, USA) or anti-LCN2 antibody (26991-1-AP, Proteintech, China) was pre-incubated with Protein G Magnetic Beads (HY-K020, MCE, USA) at ambient temperature for 10 min under gentle rotation. The antibody-Protein G bead complex was subsequently incubated with the protein lysate overnight at 4 °C with continuous rotation. Subsequently, the beads underwent five washing cycles and elution utilizing 0.1 M glycine buffer (pH 2.5). The resulting immunoprecipitated complexes were analyzed by silver staining, mass spectrometry, and western blotting.

According to the procedure of mass spectrometry, the samples were reduced and alkylated, then hydrolyzed with trypsin (mass ratio 1:50) at 37 °C for 20 hours, the hydrolysate was then desalted, freeze-dried, redissolved in 0.1% formic acid solution, and stored at -20 °C for use. A chromatographic column was equilibrated in 95% Liquid A (an aqueous solution of 0.1% formic acid) and the sample was then loaded into the Trap column by an automatic sampler. The mass charge ratio of polypeptides and polypeptide fragments was collected as follows: 20 fragments were collected after each full scan (MS2 scan). Mass spectrometry test raw files were retrieved from the corresponding database using Proteome Discoverer 1.4 software and then identified.

### Immunofluorescence (IF) staining

HCC cells were fixed with 4% paraformaldehyde for 30 min at ambient temperature, followed by triple rinsing with PBS for 5 min per wash. Subsequently, cells were permeabilized with PBS containing 0.2% Triton X-100 and blocked with 3% BSA (4240GR, BioFroxx, Germany) for a duration of 2 h at ambient temperature. Incubation with primary antibodies was conducted overnight at 4 °C. Afterward, the cells underwent another series of PBS washes (three times, 5 min each) before being exposed to fluorescence-conjugated secondary antibodies for 1 h at ambient temperature. After a final PBS rinse (three times, 5 min each), cells were counterstained with DAPI (D9542-5, Sigma, USA) for 5 min at ambient temperature. The primary antibodies employed included: anti-ZCCHC4 (HPA035575, Sigma, USA), anti-TMEM97 (26444-1-AP, Proteintech, China), anti-LCN2 (sc-518095, Santa Cruz, USA), anti-E-Cadherin (3195S, CST, USA), anti-Claudin-1 (13995S, CST, USA), and anti-Vimentin (5741S, CST, USA) antibodies.

### Luciferase reporter assays

HCC cells were co-transfected with firefly luciferase reporter plasmids (pTMEM97, pHMGCR, pSQLE, pFASN, pSCD, or the empty vector pGL3-Basic) and either shZCCHC4, shScramble, ZCCHC4 overexpression plasmid, or empty vector control plasmid using Lipofectamine 3000 (L3000, Thermo, USA) transfection reagent in accordance with the manufacturer's standard protocol. Following a 48-hour incubation post-transfection, luciferase activity was assessed utilizing the Dual-Luciferase Reporter Assay System (E1910, Promega, USA).

### Transwell migration and invasion assays

Transwell chambers (pore size 8 µm, 3422, Corning, USA) pre-coated with Matrigel were employed for the invasion assay, whereas uncoated chambers served for the migration assay. Briefly, 200 μl of serum-free medium containing suspended cells at a density of 8×10⁴ to 20×10⁴ cells/well was seeded into the upper compartment of each chamber, and the lower compartment was filled with complete medium supplemented with 10% fetal bovine serum (FBS). After 24 h of incubation at 37 °C with 5% CO₂, non-migrated/non-invaded cells remaining on the upper surface of the membrane were carefully removed with a cotton swab. Cells that had migrated or invaded to the lower surface of the membrane were fixed with 4% paraformaldehyde solution, followed by staining with 0.1% crystal violet staining solution. Finally, the number of stained cells was counted in five randomly selected microscopic fields for each chamber, and the average value was calculated for statistical analysis.

### Wound-healing assay

HCC cells were seeded into 6-well plates at a density of 2×10⁵ cells per well. When the cell monolayer reached approximately 95% confluence, a linear scratch wound was created using a sterile 200 μl pipette tip along a straight ruler to ensure consistency in wound width. Detached cells and debris were then eliminated by rinsing with PBS. Cells bearing the linear wound were maintained in serum-free medium at 37 °C in a humidified incubator with 5% CO₂. The wound area was examined and imaged under a microscope at 0 h and 48 h post-scratch. The wound area was quantified using ImageJ software, and the relative wound closure rate was calculated for statistical analysis.

### Statistical analysis

All statistical analyses were conducted using SPSS 23.0 and GraphPad Prism 9.3.1. IHC scores were presented as medians, whereas all other experimental data were denoted as mean ± standard error of the mean (s.e.m.). The association between ZCCHC4 expression patterns and clinical characteristics in HCC patients was evaluated by chi-square test. Survival curves were plotted using the Kaplan-Meier method, and differences in survival outcomes were compared by log-rank test. The Cox proportional hazards model was employed to identify independent prognostic factors through univariate and multivariate analyses. The correlations between two continuous variables were assessed by simple linear regression. Comparisons between two independent groups were performed using Student's t-test, whereas multiple group comparisons were analyzed by one-way analysis of variance (ANOVA). All statistical tests were two-tailed with a 95% confidence interval, and a P value of < 0.05 was considered statistically significant.

## Results

### ZCCHC4 is essential for HCC metastasis *in vitro* and *in vivo*

**Figure 1:**
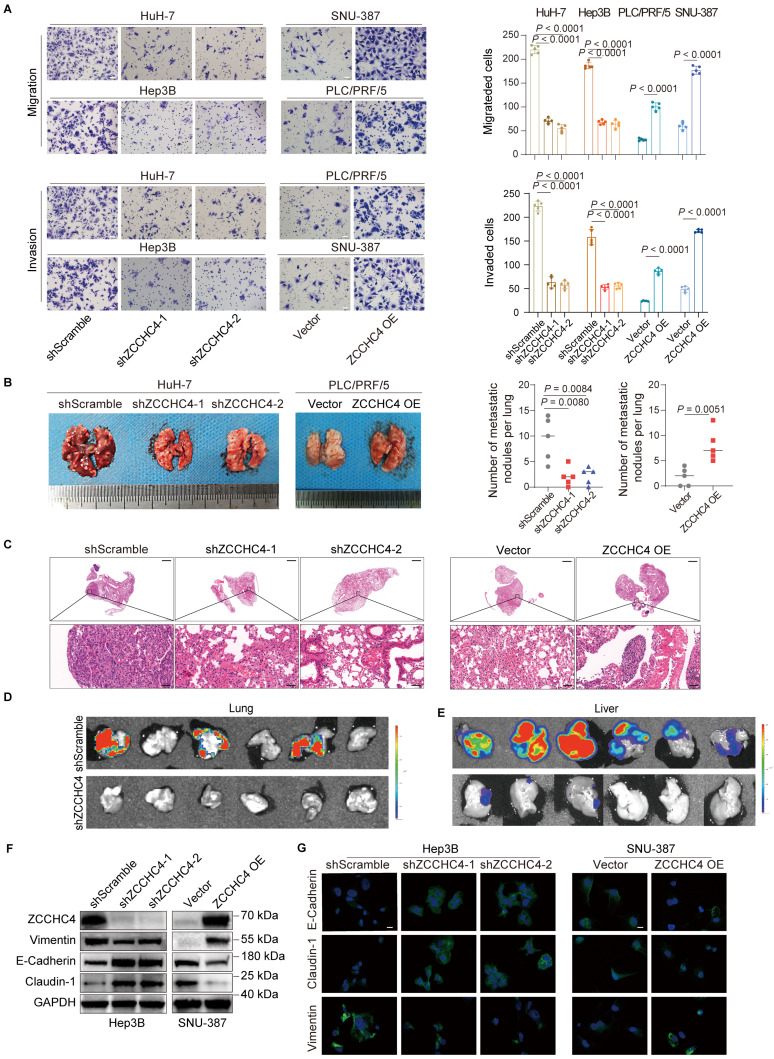
** ZCCHC4 is required for HCC metastasis and EMT. A.** Transwell migration and invasion assays with or without Matrigel coating were performed to evaluate the effect of ZCCHC4 on HCC cell migration and invasion. Scale bar, 100 μm. **B.** Representative *in vivo* images (left panel) and quantification of metastatic lung nodules (right panel) illustrating the effect of ZCCHC4 on HCC lung metastasis. **C.** Hematoxylin and eosin (HE) staining of mouse lung tissues from different groups: negative control, ZCCHC4 knockdown, ZCCHC4 overexpression vector control, and ZCCHC4 overexpression, to detect metastatic HCC cells. Scale bars = 1,000 μm (upper panels) and 50 μm (lower panels). **D.** Effect of ZCCHC4 on lung metastasis in an orthotopic HCC mouse model. **E.** Effect of ZCCHC4 on tumor growth in the orthotopic HCC mouse model. **F.** Western blotting analysis was conducted to examine the effect of ZCCHC4 on the expression levels of the EMT marker Vimentin and the mesenchymal-epithelial transition (MET) markers E-Cadherin and Claudin-1. **G.** Immunofluorescence (IF) staining showing the expression patterns of Vimentin, Claudin-1, and E-Cadherin in HCC cells with altered ZCCHC4 expression. Scale bar, = 100 μm. Error bars represent means ± standard deviation (SD). Statistical significance was determined using a two-tailed unpaired Student's t-test (for panels A and B).

To investigate the role of ZCCHC4 in HCC metastasis, we first assessed its expression in seven HCC cell lines and the immortalized hepatocyte line MIHA. ZCCHC4 was upregulated in HCC cells at both mRNA and protein levels (Figure S1A, S1B). We then knocked down ZCCHC4 in HuH-7 and Hep3B cells (high endogenous expression) and overexpressed it in PLC/PRF/5 and SNU-387 cells (low endogenous expression), with efficiency confirmed by WB (Figure S1C). Functional assays showed that ZCCHC4 knockdown inhibited migration, invasion, and wound healing, whereas its overexpression enhanced these activities (Figure 1A, S1D). In a lung metastasis model, ZCCHC4 silencing reduced metastatic burden, while overexpression increased it, as confirmed by H&E staining (Figure 1B, 1C, S1E). Consistent results were obtained in the orthotopic HCC model, where ZCCHC4 knockdown led to substantial suppression of lung metastasis (Figure 1D). ZCCHC4 knockdown also inhibited tumor growth (Figure 1E), consistent with prior reports 22. Since EMT is crucial for metastasis, we examined EMT markers. ZCCHC4 knockdown decreased Vimentin and increased E-cadherin and Claudin-1 expression *in vitro* and *in vivo*, while overexpression had the opposite effect (Figure 1F, 1G, S1F-S1H). Angiogenesis, an essential event for distant metastasis, assessed by tube formation, was unaffected by ZCCHC4 (Figure S1I). Prior research has shown that cancer stem cells (CSCs) facilitate tumor relapse and metastasis 26. Among liver CSC markers, CD24, CD13, and EpCAM are commonly used 27. Flow cytometry revealed no change in CSC markers upon ZCCHC4 silencing (Figure S1J). Together, these findings demonstrate that ZCCHC4 promotes HCC metastasis primarily by regulating EMT.

### ZCCHC4 directly activates the genes encoding the rate-limiting enzymes for cholesterol and fatty acid biosynthesis and regulates the transcription of TMEM97

**Figure 2:**
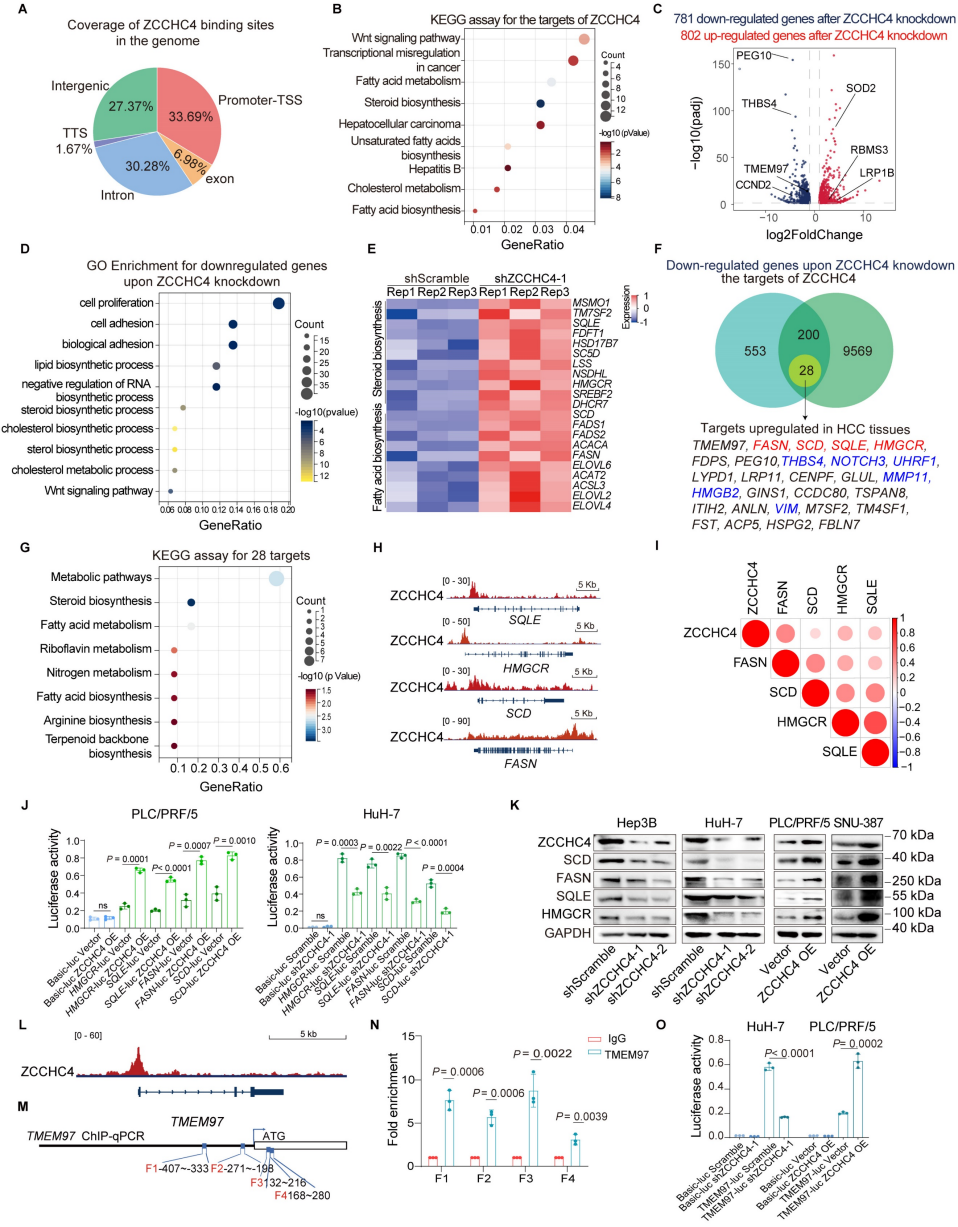
** ZCCHC4 directly activates the genes encoding the rate-limiting enzymes for cholesterol and fatty acid biosynthesis and regulates the transcription of TMEM97. A.** Genomic distribution of ZCCHC4 binding sites. **B.** KEGG pathway enrichment analysis of ZCCHC4 target genes. **C.** Volcano plot illustrating differentially expressed genes (DEGs) in HCC cells transduced with shScramble or ZCCHC4 shRNA. **D.** Gene Ontology (GO) enrichment analysis of downregulated genes upon ZCCHC4 knockdown. **E.** Heatmap showing the expression profiles of genes involved in cholesterol and fatty acid biosynthesis in HCC cells transduced with shScramble or ZCCHC4 shRNA. **F.** Venn diagram depicting the overlap among ZCCHC4 target genes, genes downregulated by ZCCHC4 knockdown, and genes upregulated in HCC tissues. **G.** KEGG pathway enrichment analysis of the “core ZCCHC4 target gene set”. **H.** Meta tracks showing ZCCHC4 CUT&Tag signal occupancy at the promoter regions of SQLE, HMGCR, FASN, and SCD. **I.** Pearson correlation analysis between ZCCHC4 mRNA expression and the mRNA levels of genes encoding rate-limiting enzymes of cholesterol or fatty acid synthesis. **J.** Luciferase reporter assay to evaluate the effect of ZCCHC4 on the transcriptional activity of genes encoding rate-limiting enzymes of cholesterol and fatty acid synthesis. **K.** Western blotting analysis to determine the effect of ZCCHC4 on the protein expression levels of SQLE, HMGCR, SCD, and FASN. **L.** Meta tracks showing ZCCHC4 CUT&Tag signal occupancy at the promoter regions of TMEM97. **M-N.** Chromatin immunoprecipitation quantitative polymerase chain reaction (ChIP-qPCR) analysis to verify the binding of ZCCHC4 to the promoter sequence of TMEM97. **O.** Luciferase reporter assay to assess the effect of ZCCHC4 on TMEM97 transcriptional activity. Error bars represent the means ± SD. Statistical significance was determined using a two-tailed unpaired Student's t-test (for panels J, N and O).

Previous studies have reported that ZCCHC4 is located in both the cytoplasm and nucleus 22. However, our IF staining results revealed that ZCCHC4 predominantly localizes in the nucleus of HCC cells (Figure S2A). ZCCHC4 contains nucleic acid-recognition and CCHC zinc-finger domains, suggesting its potential role as a transcription factor that activates genes promoting tumor metastasis and EMT. To identify its direct targets, we performed CUT & Tag analysis (Figure 2A). KEGG pathway analysis of ZCCHC4-bound targets showed associations with hepatocellular carcinoma, lipid metabolism, and the Wnt pathway (Figure 2B). Similarly, GO analysis of genes downregulated following ZCCHC4 knockdown revealed enrichment in pathways related to lipid metabolism, cell adhesion and migration, and the Wnt pathway (Figure 2C and 2D). The GSEA analysis of RNA-seq showed the genes regulated by ZCCHC4 were significantly related to EMT (Figure S2B)**.**

Previous studies have shown that HCC metastasis is closely related to lipid metabolism reprogramming, Wnt pathway activation and EMT induction. Key genes involved in cholesterol and fatty acid biosynthesis were significantly downregulated upon ZCCHC4 knockdown, highlighting its pivotal role in lipid metabolism (Figure 2E). To identify potential direct targets of ZCCHC4 involved in HCC metastasis, we then integrated three criteria to define a core set of direct ZCCHC4 target genes: promoter binding by CUT&Tag, downregulation after ZCCHC4 knockdown, and upregulation in HCC tissues. This yielded 28 genes, including known metastasis promoters such as VIM, THBS4, MMP11, HMGB2, and NOTCH3 (Figure 2F) 28-30. This core gene set was highly enriched in cholesterol and fatty acid biosynthesis pathways (Figure 2G), and included the key metabolic enzymes HMGCR, SQLE, FASN, and SCD (Figures 2F and 2H). HMGCR and SQLE serve as rate-limiting enzymes in cholesterol biosynthetic pathways 31, whereas FASN and SCD are recognized as key regulators in fatty acid biosynthesis 32. Correlation analysis based on RNA-seq data from the Cancer Genome Atlas (TCGA) database demonstrated a significant positive relationship between ZCCHC4 transcript levels and the expression of HMGCR, SQLE, FASN, and SCD (Figure 2I). luciferase reporter assay showed that ZCCHC4 knockdown markedly attenuated promoter activity for HMGCR, SCD, FASN, and SQLE. In contrast, ZCCHC4 overexpression produced the opposite effect (Figure 2J and S2C). Consistent with these findings, western blotting showed corresponding changes in their protein levels (Figure 2K).

Additionally, the Wnt pathway activator TMEM97 was identified as a core ZCCHC4 target (Figure 2F and 2L). To examine whether TMEM97 transcription is regulated by ZCCHC4, ChIP-qPCR analysis was conducted, confirming ZCCHC4 binding to the *TMEM97* promoter (Figure 2M-2N), and luciferase assays demonstrated ZCCHC4-dependent transcriptional activation (Figure 2O), which was consistent with protein-level changes (Figure S4A). In summary, ZCCHC4 functions as a transcriptional activator of key lipid metabolism genes and TMEM97 to promote HCC progression.

### ZCCHC4 promotes metastasis by regulating lipid biosynthesis in HCC

**Figure 3:**
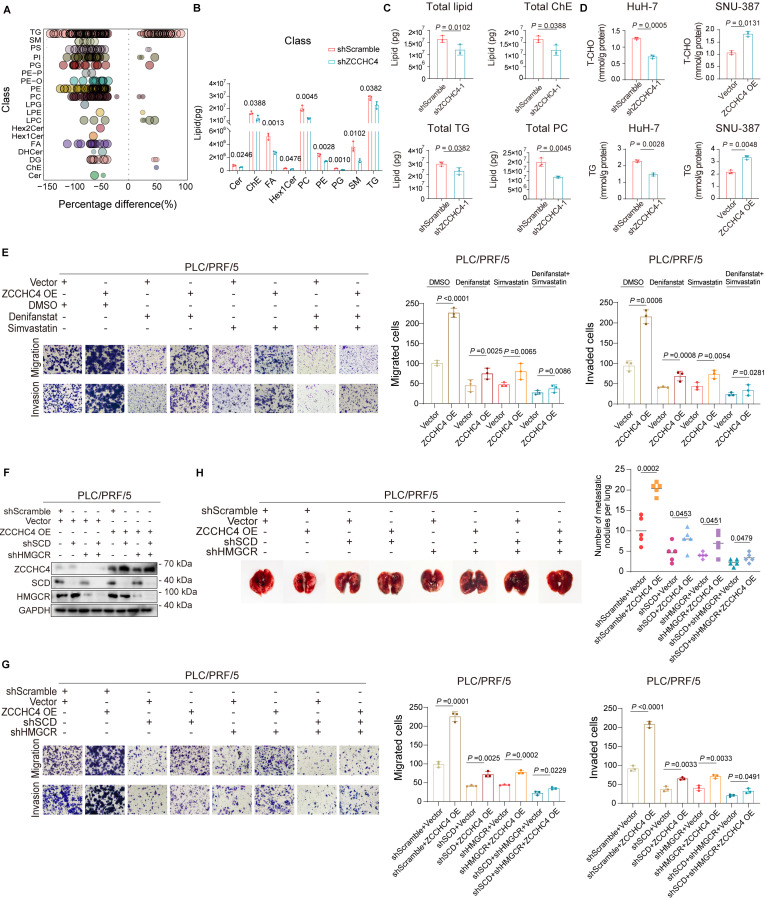
** ZCCHC4 promotes HCC metastasis by regulating lipid biosynthesis. A and B.** Quantification of total levels of cholesterol esters (ChE), fatty acid (FA), phosphatidylcholine (PC), phosphatidylethanolamine (PE), phosphatidylglycerol (PG), sphingomyelin (SM), monosaccharide cerebroside (Hex1Cer), and triglycerides (TG) in HCC cells transduced with shScramble or ZCCHC4 shRNA.** C.** Determination of intracellular levels of total lipid, total ChE, total TG and total PC in HCC cells transduced with shScramble or ZCCHC4 shRNA. **D.** Intracellular quantification of total cholesterol (T-CHO) and TG in HCC cells transduced with shScramble, ZCCHC4 shRNA, empty vector, or ZCCHC4 OE. **E.** Transwell migration and invasion assays (using uncoated or Matrigel-coated inserts, respectively) to evaluate the effects of fatty acid synthesis inhibitor and/or cholesterol synthesis inhibitor treatment on the migratory and invasive capacities of ZCCHC4-overexpressing HCC cells. **F.** Western blotting analysis was performed to examine the expression of SCD and HMGCR in ZCCHC4-overexpressing HCC cells transduced with shRNAs targeting SCD and/or HMGCR. **G.** Transwell migration and invasion assays (Matrigel-uncoated and Matrigel-coated inserts, respectively) were performed to evaluate the effect of SCD and/or HMGCR knockdown on the migratory and invasive capacities of ZCCHC4-overexpressing HCC cells. **H.** Representative *in vivo* images (left panel) and quantification of metastatic lung nodules (Right panel) showing the effect of SCD and/or HMGCR knockdown on the metastatic potential of ZCCHC4-overexpressing HCC cells. Error bars represent the mean ± SD. Statistical significance was determined using Student's t-test. **(A-E, G-H)**.

Lipid accumulation serves as an energy source that fuels metastasis, a process driven by the upregulation of cholesterol and fatty acid biosynthesis in HCC 7. To determine whether ZCCHC4 facilitates this accumulation, we performed targeted lipidomics. ZCCHC4 knockdown significantly reduced the biosynthesis of multiple lipid species, including triglycerides (TG), cholesterol esters (ChE), phosphatidylcholine (PC), sphingolipids, and phosphoglycerides (Figure 3A and 3B, S3A-3C). Intracellular levels of total cholesterol (T-CHO) and TG were correspondingly decreased upon knockdown and increased upon overexpression (Figure 3C and 3D). Consistently, Nile red staining showed that lipid droplet formation was reduced by ZCCHC4 silencing and enhanced by its overexpression (Figure S3D). These results indicate that ZCCHC4 drives lipid synthesis in HCC through the orchestration of lipid metabolic reprogramming.

To clarify whether ZCCHC4 promotes metastasis by regulating lipid reprograming, we performed rescue experiments. Inhibitors of fatty acid or cholesterol synthesis each partially reversed the pro-migratory effect of ZCCHC4 overexpression, with stronger inhibition upon combined treatment (Figure 3E). We then knocked down the key enzymes SCD or HMGCR in ZCCHC4-overexpressing cells (Figure 3F). Loss of either SCD or HMGCR attenuated the pro-migratory, pro-invasive, and pro-metastatic effects induced by ZCCHC4, with the most pronounced suppression observed upon their combined knockdown (Figure 3G, 3H and S3E). Collectively, these findings demonstrate that ZCCHC4 promotes HCC metastasis by facilitating fatty acid and cholesterol synthesis, underscoring the critical role of lipid metabolic reprogramming in ZCCHC4-mediated progression.

### ZCCHC4 activates the Wnt pathway by enhancing the transcription of TMEM97 to promote metastasis of HCC

**Figure 4:**
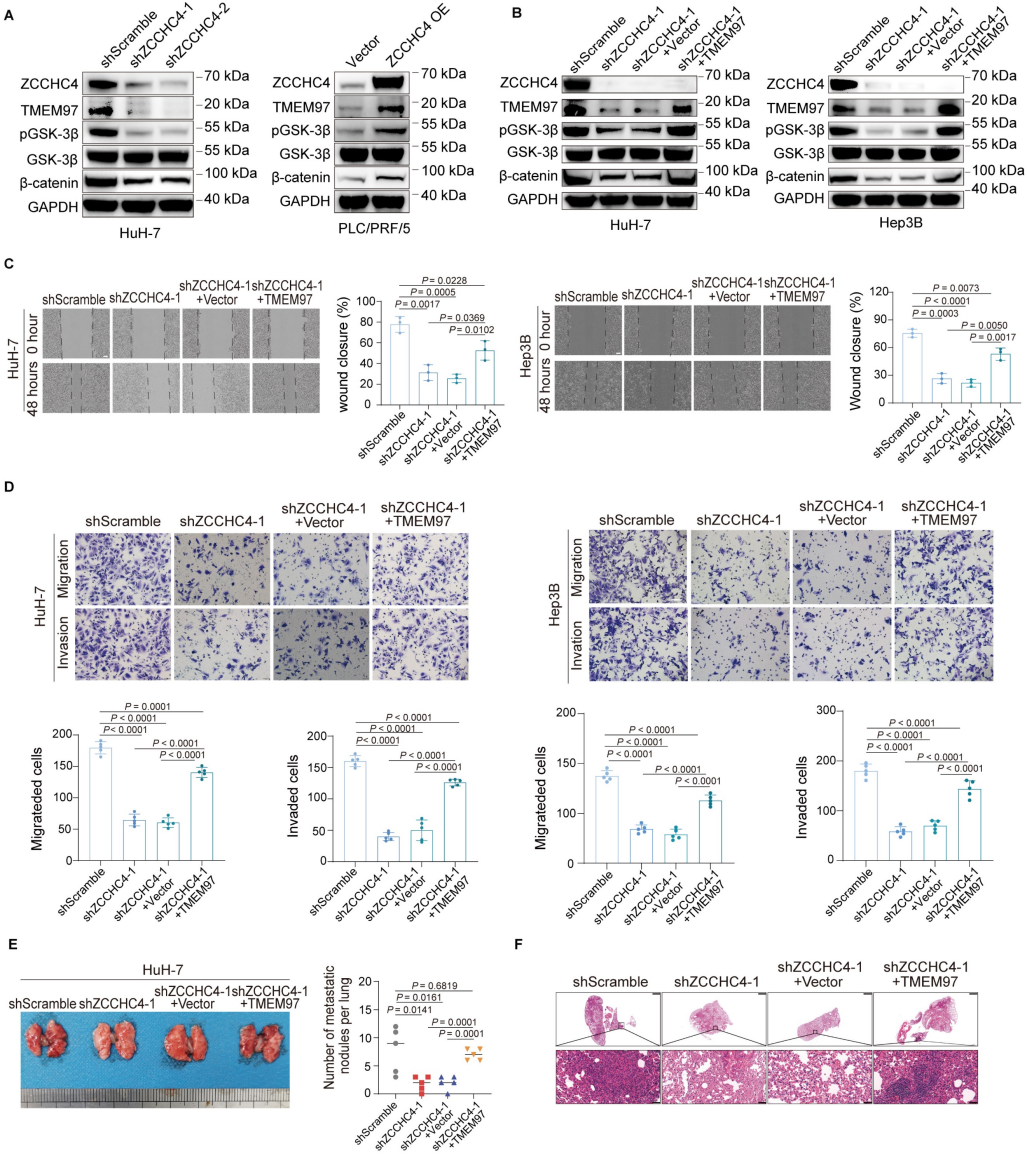
** ZCCHC4 activates the Wnt pathway by enhancing the transcription of TMEM97 to promote metastasis of HCC. A.** Western blotting analysis showing the effect of ZCCHC4 on the expression of TMEM97, GSK3β, phospho-GSK3β, and β-catenin. **B.** Western blotting analysis showing the effect of TMEM97 overexpression on the expression of GSK3β, phospho-GSK3β, and β-catenin in HCC cells transduced with shScramble or ZCCHC4 shRNA. **C.** Wound healing assay showing the effect of TMEM97 overexpression on migration in HCC cells transduced with shScramble or ZCCHC4 shRNA. Scale bar, 100 μm. **D.** Transwell assay (Matrigel-uncoated or Matrigel-coated) showing the effect of TMEM97 overexpression on the migration and invasion abilities of HCC cells transduced with shScramble or ZCCHC4 shRNA. Scale bar, 100 μm. **E** Representative *in vivo* images (left panel) and quantification of metastatic lung nodules (right panel) illustrating the effect of TMEM97 overexpression in HCC cells transduced with shScramble or ZCCHC4 shRNA. **F.** H&E staining of lung tissues from mice injected with HCC cells of different groups: shScramble, ZCCHC4 knockdown, ZCCHC4 knockdown plus empty vector, and ZCCHC4 knockdown plus TMEM97 overexpression. Scale bar, 1000 μm (upper panels); scale bar, 50 μm (lower panels). Error bars represent the means ± SD. Statistical significance was determined using two-tailed unpaired Student's t-test for panels**(C-E)**.

Previous studies have demonstrated that the interaction between TMEM97 and the intracellular domain of LRP6 promotes the recruitment of CK1δ/ε, which enhances the activity of Wnt pathway and thus promotes metastasis in breast cancer 33. Subsequently, the effect of ZCCHC4 on TMEM97 expression and Wnt signaling pathway activity was evaluated. Western blotting showed that ZCCHC4 knockdown reduced the levels of TMEM97, phospho-GSK3β, and β-catenin, while its overexpression increased them (Figure 4A and S4A). This suggests ZCCHC4 activates Wnt signaling. To determine if this occurs through TMEM97, we overexpressed TMEM97 in ZCCHC4-knockdown cells (Figure S4B). This restored phospho-GSK3β and β-catenin levels (Figure 4B), indicating that ZCCHC4 facilitates Wnt pathway activation through transcriptional upregulation of TMEM97.

Conversely, inhibiting the Wnt pathway or TMEM97 expression did not affect ZCCHC4 expression, indicating a unidirectional regulation (Figure S4C). The Wnt signaling cascade functions as an essential mediator in HCC proliferation and metastatic dissemination 34. To assess whether ZCCHC4 facilitates HCC metastasis via TMEM97-mediated Wnt pathway activation, TMEM97 was overexpressed in cells with ZCCHC4 knockdown, resulting in a partial restoration of migratory, invasive and metastatic capabilities *in vitro* and *in vivo* (Figure 4C-4F and S4D). Given previous findings that TMEM97 promotes HCC growth by activating Wnt/GSK-3β/β-catenin pathway 35, the potential contribution of TMEM97 to this process was further investigated. TMEM97 overexpression also reversed the suppression of cell proliferation and tumor growth caused by ZCCHC4 knockdown (Figure S4E-S4G). In summary, ZCCHC4 promotes HCC growth and metastasis by transcriptionally upregulating TMEM97, which in turn mediates the aforementioned biological effects through activation of the Wnt/β-catenin pathway.

ZCCHC4 promotes HCC metastasis primarily through two pathways: lipid metabolism and TMEM97-mediated Wnt signaling. To compare their relative contributions, rescue experiments were performed. Inhibitors targeting either pathway partially reversed the pro-migratory and pro-invasive effects induced by ZCCHC4 overexpression (Figure S4H). Notably, combined inhibition of lipid synthesis exerted a more pronounced suppressive effect on ZCCHC4 overexpression-mediated cell migration and invasion: ZCCHC4 overexpression only enhanced the migratory and invasive capacities of HCC cells by approximately 41% in the combined inhibitor group, a level significantly lower than the 58% observed in the single Wnt signaling inhibitor group (Figure S4H). These results indicate that ZCCHC4 displays a greater dependence on the lipid metabolic axis to drive cell migration and invasion. Moreover, we further examined potential crosstalk between these pathways. Although ZCCHC4 overexpression upregulated TMEM97, SCD, and HMGCR, knockdown of SCD or HMGCR did not affect TMEM97 expression or prevent its upregulation by ZCCHC4 (Figure S4I). These results demonstrate that SCD, HMGCR, and TMEM97 function as independent downstream targets of ZCCHC4, with no intrinsic interaction between the lipid metabolic and TMEM97-mediated branches.

### TMEM97 promotes EMT in HCC through cytoplasmic sequestration of LCN2 and subsequent Twist1 activation

**Figure 5:**
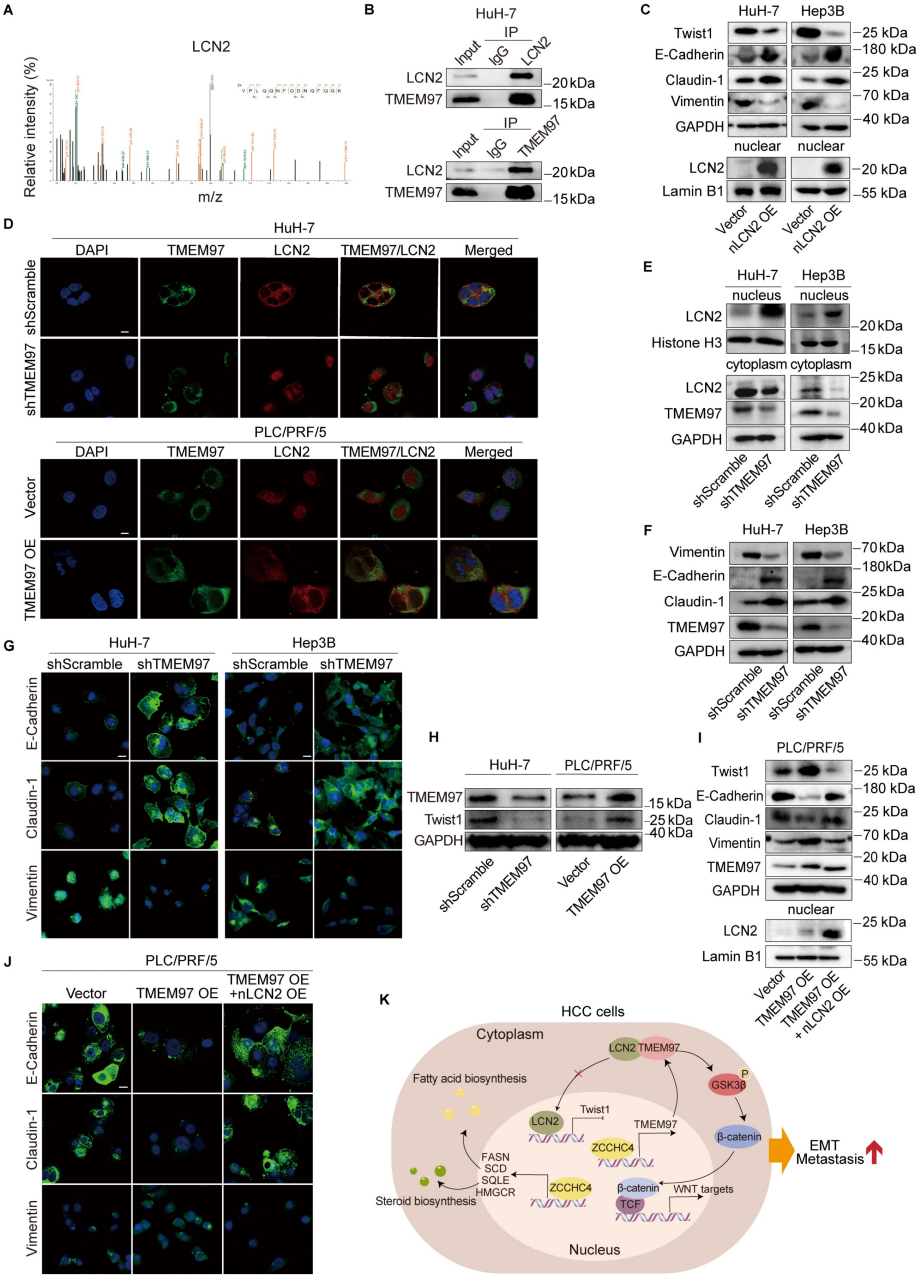
** TMEM97 promotes EMT in HCC through cytoplasmic sequestration of LCN2 and subsequent Twist1 activation. A.** Identification of the interaction between LCN2 and TMEM97 by mass spectrometry. **B.** CoIP analysis verifying the interaction of TMEM97 with LCN2 in high-metastatic HCC cells. **C.** Western blotting analysis showing the effect of nuclear LCN2 overexpression on EMT associated proteins. **D.** IF staining illustrating the subcellular localization of LCN2 in HCC cells transduced with TMEM97 shRNA or TMEM97 overexpression vector. Scale bar, 100 μm. **E.** Western blotting detecting the subcellular distribution of LCN2 in high-metastatic HCC cells with TMEM97 knockdown. **F.** Western blotting analysis of the expression levels of EMT markers (E-cadherin, Vimentin, and Claudin-1) in HCC cells with TMEM97 knockdown. **G.** IF staining showing the expression of EMT markers (E-cadherin, Vimentin, and Claudin-1) in HCC cells with TMEM97 knockdown. Scale bar, 100μm. **H.** Western blotting analysis showing the regulatory effect of TMEM97 on expression of Twist1. **I.** Western blotting assay evaluating the effect of nuclear LCN2 and TMEM97 co-overexpression on the expression of EMT-associated proteins. **J.** IF staining showing the effect of nuclear LCN2 and TMEM97 co-overexpression on the expression of EMT-associated proteins. Scale bar, 100 μm. **K.** Proposed working model illustrating the role of ZCCHC4 in HCC metastasis and EMT.

To further investigate how TMEM97 promotes HCC metastasis, we performed co-immunoprecipitation coupled with mass spectrometry (CoIP-MS) and identified LCN2 as a novel binding partner of TMEM97 (Figure 5A). This interaction was subsequently validated by reciprocal CoIP assays in HCC cells (Figure 5B and S5A). Prior studies have demonstrated that the nuclear translocation of LCN2 inhibits EMT and impedes HCC metastasis 36. In contrast, LCN2 localized in the cytoplasm or secreted extracellularly, has been reported to facilitate iron depletion, thereby reducing the susceptibility of HCC cells to ferroptosis inducers, suggesting that the functional role of LCN2 is dependent on its subcellular distribution 37. Consistently, we found that overexpression of nuclear-targeted LCN2 downregulated the expression levels of Twist1 and Vimentin, while upregulating E-Cadherin and Claudin-1 (Figure 5C). To examine whether TMEM97 regulates LCN2 localization, we performed IF staining. In mesenchymal-like HuH-7 cells, LCN2 co-localized with TMEM97 in the cytoplasm, whereas in epithelial-like PLC/PRF/5 cells, LCN2 was mainly nuclear (Figure 5D). TMEM97 knockdown in HuH-7 cells promoted LCN2 nuclear translocation, while TMEM97 overexpression in PLC/PRF/5 cells enhanced its cytoplasmic retention (Figure 5D). These results were further supported by western blotting, which showed that TMEM97 knockdown facilitated nuclear accumulation of LCN2 during mesenchymal-to-epithelial transition (MET) (Figure 5E-5G). Together, these data indicate that TMEM97 modulates the subcellular distribution of LCN2. Furthermore, we examined whether ZCCHC4, which transcriptionally upregulates TMEM97, affects LCN2 localization. Consistent with the effects of modulating TMEM97, ZCCHC4 knockdown promoted LCN2 nuclear import, while its overexpression enhanced LCN2 cytoplasmic retention (Figure S5C). These results indicate that ZCCHC4 regulates LCN2 trafficking by controlling TMEM97 expression.

Nuclear LCN2 is known to suppress HCC metastasis and EMT by inhibiting Twist1 transcription 36. In line with this, we found that TMEM97 knockdown decreased Twist1 expression, while TMEM97 overexpression increased it (Figure 5H). Co-overexpression of TMEM97 and nuclear LCN2 in epithelial-like HCC cells reversed the TMEM97-mediated upregulation of Twist1 and attenuated EMT induction (Figure 5I and 5J). Consistent with this, transwell migration and invasion assays showed that co-expression of nuclear LCN2 partially counteracted the pro-migratory and pro-invasive effects of TMEM97 overexpression (Figure S5B). Collectively, our results establish that ZCCHC4 promotes HCC EMT and metastasis by transcriptionally activating TMEM97, which in turn inhibits LCN2 nuclear translocation to enhance Twist1 activity. In summary, ZCCHC4 promotes HCC metastasis via a multi-pronged mechanism: it transcriptionally upregulates lipid synthesis genes, activates the Wnt pathway through TMEM97, and inhibits the nuclear translocation of LCN2, thereby potentiating Twist1 expression (Figure 5K).

### High ZCCHC4 expression linked to lipid metabolism, TMEM97 upregulation, and poor prognosis in HCC

**Figure 6:**
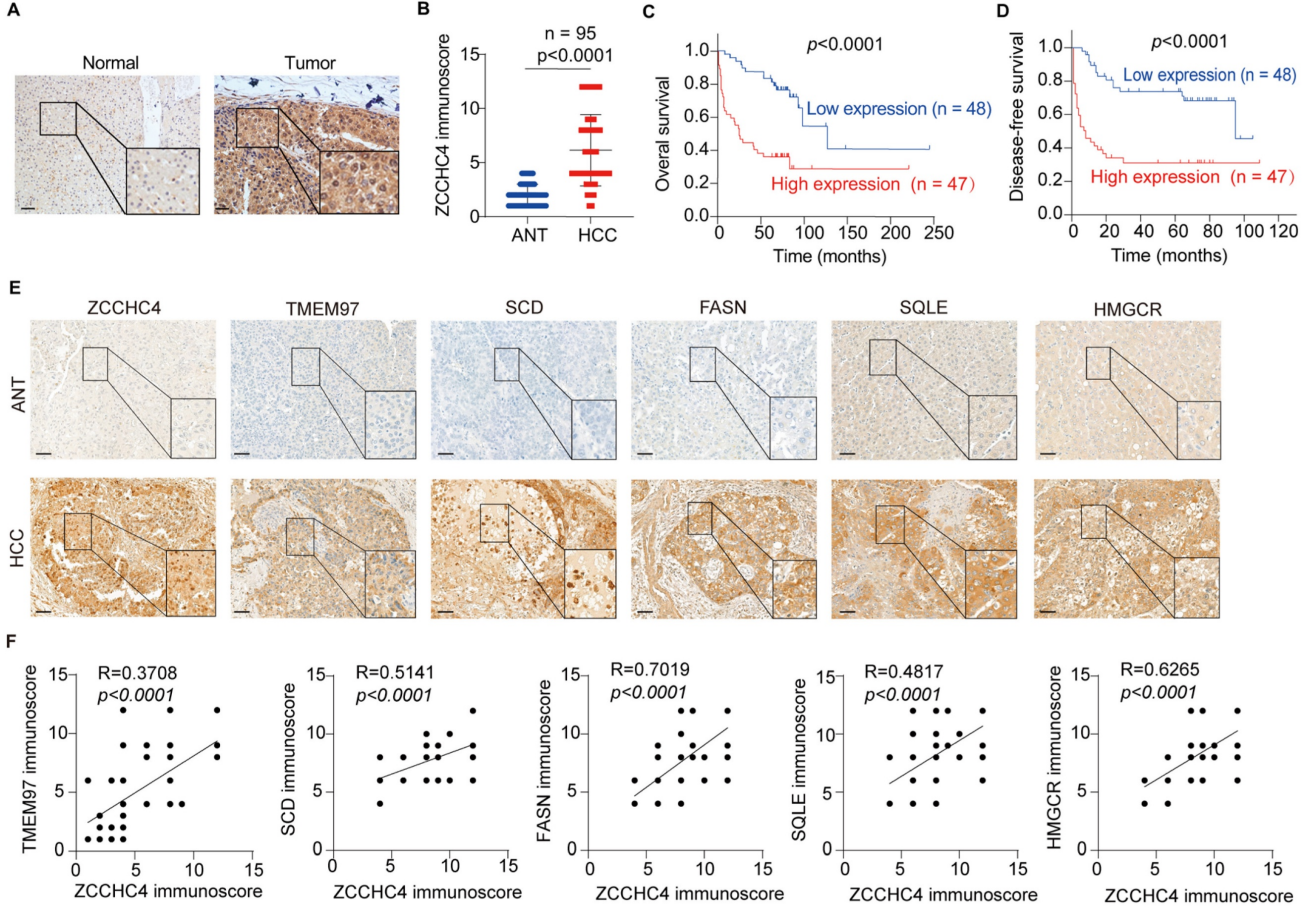
** High ZCCHC4 expression linked to lipid metabolism, TMEM97 upregulation, and poor prognosis in HCC. A.** Representative IHC micrographs showing ZCCHC4 expression in HCC tissues and their adjacent normal liver tissues. Scale bar, 100 μm. **B.** ZCCHC4 expression in primary HCC tissues and adjacent normal liver tissues as determined by IHC. **C-D.** Kaplan-Meier survival curves with log-rank test analyzing the association of high and low ZCCHC4 expression with overall survival (C) and disease-free survival (D) in HCC patients. **E-F.** Correlation analysis of ZCCHC4 expression with the expression levels of lipid metabolic rate-limiting enzymes (SQLE, HMGCR, SCD, FASN) and TMEM97 in HCC tissues. Scale bars, 100 μm (uppe panels) and 50 μm (lower panels). Error bars represent means ± SD. Statistical significance was determined using two-tailed unpaired Student's t-test (B), log-rank test (C, D), and simple linear regression analysis (F).

We determined ZCCHC4 expression in HCC tissues and found that ZCCHC4 was evaluated in 95 matched HCC and adjacent non-tumorous liver samples (Table S1 and Figure 6A-6B). Elevated ZCCHC4 levels were significantly associated with aggressive clinicopathological features, including T stage, TNM stage, encapsulation invasion, and vascular invasion (Table S2). Kaplan-Meier survival analysis showed that patients with low ZCCHC4 expression had significantly longer overall survival and disease-free survival compared to those with high expression (Figure 6C, 6D). Furthermore, univariate and multivariate Cox regression analyses identified ZCCHC4 as an independent prognostic factor for HCC (Tables S3 and S4). These results demonstrate that high ZCCHC4 expression correlates with unfavorable clinicopathological features and poor survival, supporting its potential as an independent prognostic biomarker in HCC.

In addition, HMGCR, SQLE, FASN, SCD and TMEM97 were also significantly overexpressed in HCC tissues (Figure 6E). Correlation analysis showed that ZCCHC4 expression was positively associated with these key lipid metabolic enzymes (HMGCR: R² = 0.6265; SQLE: R² = 0.4817; FASN: R² = 0.7019; SCD: R² = 0.5141; all p < 0.0001; Figure 6F) as well as with TMEM97 (R² = 0.3708, p < 0.0001; Figure 6F). Taken together, these results demonstrate that ZCCHC4 expression is positively correlated with the levels of lipid metabolism enzymes and TMEM97 in HCC, further supporting its link to poor clinical prognosis.

## Discussion

Recent studies have identified ZCCHC4 as an m^6^A methyltransferase that promotes HCC tumor growth by modifying 28S rRNA 22. Independently, ZCCHC4 also functions as an RNA-binding protein that suppresses DNA damage-induced apoptosis through competitive binding with lncRNA AL133467.2 24. In this work, we uncover a distinct, transcription-regulatory role of ZCCHC4: it drives EMT and metastasis in HCC by directly activating genes involved in cholesterol/fatty acid biosynthesis, the TMEM97-mediated Wnt pathway, and the TMEM97/LCN2/Twist1 axis. These findings significantly expand the functional repertoire of ZCCHC4 in HCC pathogenesis.

Dysregulated lipid metabolism supplies cancer cells with essential nutrients, signaling molecules, and membrane components that support tumor initiation, progression, and metastasis. Elevated levels of circulating cholesterol and fatty acids are linked to poor prognosis in cancer and have been shown to promote HCC metastasis and induce EMT 38-41. Nevertheless, the role of ZCCHC4 in lipid metabolic reprogramming remains unexplored. In this study, multi-omics analyses indicated that ZCCHC4 regulates lipid metabolism, specifically cholesterol and fatty acid biosynthesis. Mechanistically, ZCCHC4 transcriptionally activates key lipid-synthesis enzymes: HMGCR and SQLE (cholesterol biosynthesis) and FASN and SCD (fatty acid biosynthesis). Previous studies have established that SQLE and HMGCR are transcriptionally regulated by SREBP2, while SREBP1 modulates the expression of FASN and SCD 42. In addition, HMGCR has also been reported to be transcriptionally activated by XBP1 and ARID1A 43, 44. Our work reveals that ZCCHC4 can directly and simultaneously upregulate all four, expanding the understanding of transcriptional control over lipogenesis in cancer. Collectively, these results establish ZCCHC4 as a central driver of lipid metabolic reprogramming in HCC and highlight its potential as a therapeutic target.

Beyond its role in lipogenesis, ZCCHC4 also directly activates other genes that contribute to HCC metastasis and EMT. For instance, THBS4, previously shown to promote HCC growth and metastasis, is transcriptionally activated by ZCCHC4 45. Vimentin, a type III intermediate filament protein and core mesenchymal marker, is strongly associated with the promotion of HCC metastasis. As a structural component of the mesenchymal cytoskeleton, it provides mechanical support and cellular plasticity. During EMT, Vimentin is markedly upregulated and serves as a key indicator of EMT 46. Our results indicate that ZCCHC4 may directly regulate Vimentin transcription by binding to its promoter. More importantly, we identified that TMEM97 is a direct target of ZCCHC4. Although TMEM97 is known to activate the Wnt pathway and support HCC growth 34, its functions in metastasis and EMT were undefined. TMEM97 enhanced HCC metastasis and Twist1 expression. Rescue experiments showed that TMEM97 overexpression reversed the suppressive effects of ZCCHC4 knockdown on invasion, migration, growth, metastasis, and Wnt signaling. Mechanistically, TMEM97 interacts with LCN2 in the cytoplasm and inhibits its nuclear import, leading to increased Twist1 expression. Our study thus establishes that TMEM97 promotes HCC progression through dual mechanisms: Wnt pathway activation and cytoplasmic retention of LCN2, offering new perspectives on its role in tumor progression.

LCN2 is implicated in tumor growth, metastasis, chemoresistance, and EMT across multiple cancer types 47, 48. But it plays controversial roles in HCC progression. On one hand, LCN2 can act as an oncogene; for example, loss of the tumor suppressor LIFR activates NF-κB signaling to upregulate LCN2, which in turn inhibits ferroptosis in HCC. Neutralizing LCN2 has been shown to enhance treatment efficacy by inducing ferroptosis 37. On the other hand, LCN2 can also function as a tumor suppressor: it has been reported to inhibit HCC proliferation, invasion, and metastasis by transcriptionally repressing the EMT transcription factor Twist1 36. In the present study, we found that LCN2 localization varies with metastatic potential: in highly metastatic HCC cells, LCN2 is predominantly cytoplasmic, whereas in cells with low metastatic ability, it is mainly nuclear. Since nuclear LCN2 directly binds to the Twist1 promoter to suppress its expression and inhibit EMT, these findings indicate that the subcellular localization of LCN2 is a key determinant of its functional outcome in HCC progression.

In summary, our findings establish ZCCHC4 as a key driver of HCC metastasis. It promotes EMT and metastatic dissemination by reprogramming lipid metabolism, activating the Wnt pathway, and sequestering LCN2 in the cytoplasm. Targeting ZCCHC4 therefore represents a promising therapeutic strategy for HCC.

## Supplementary Material

Supplementary figures and tables.
