# Supplementary material for: ZCCHC4 Orchestrates Hepatocellular Carcinoma Metastasis by Regulating Lipid biosynthesis and TMEM97/LCN2/Twist1 Pathway
Source: Int J Biol Sci. 2026 Mar 25;22(7):3600–16. doi: 10.7150/ijbs.120086 (PMC13086013; doi:10.7150/ijbs.120086)
Supplement: Supplementary file 1 — Supplementary figures and tables. [file ijbsv22p3600s1.pdf]

Supplementary Materials for

ZCCHC4 Orchestrates Hepatocellular Carcinoma Metastasis by Regulating Lipid biosynthesis and TMEM97/LCN2/Twist1 Pathway

Juan Ye[1,2,3,8#], Xinyi Wen[1,2,3,#], Ruiyang Liu[1,2,3#], Caini Huang[1,2,3,#], Zhijie Xu[1,2,3], Yi Zhan[1,2,3], Fuyuan Xu[1,2,3], Hongbin Huang[1,2,3], Chunhui Qi[1,2,3], Yao Tang[1,2,3], Peirong Li[1,2,3], Jianzhong He[4], Zhiju Zhao[5,*], Gang Lu[5,*], Fei Xiao[1,2,3,6,7,*]

[1]Department of Infectious Diseases, The Fifth Affiliated Hospital, Sun Yat-sen University, Zhuhai, Guangdong Province, China.

[2]Guangdong Provincial Engineering Research Center of Molecular Imaging, The Fifth Affiliated Hospital, Sun Yat-sen University, Zhuhai, Guangdong Province, China.

[3]Guangdong-Hong Kong-Macao University Joint of Interventional Medicine, The Fifth Affiliated Hospital, Sun Yat-sen University, Zhuhai, Guangdong Province, China.

[4]Department of Pathology, The Fifth Affiliated Hospital, Sun Yat-sen University, Zhuhai, Guangdong Province, China.

[5]Key Laboratory for Regenerative Medicine, Ministry of Education, School of Biomedical Sciences, Faculty of Medicine, The Chinese University of Hong Kong, Hong Kong SAR, China.

[6]Kashi Guangdong Institute of Science and Technology, The First People's Hospital of Kashi, Kashi, Xinjiang Uyghur Autonomous Region, China.

[7]State Key Laboratory of Anti-Infective Drug Development, School of Pharmaceutical Sciences, Sun Yat-sen University, Guangzhou, Guangdong Province, China.

[8]Department of Oncology, The Second Affiliated Hospital of Zunyi Medical University, Zunyi, Guizhou Province, China.

[#]These authors contributed equally to this work.

*Corresponding authors:

Supplementary materials and methods

Cell lines and cell culture

The human HCC cell lines HuH-7, Hep3B, PLC/PRF/5, HepG2, SK-HEP-1, SNU-387, SNU-182, as well as immortal hepatocyte MIHA (CL0469) were procured from the

Cell Resource Center of the Chinese Academy of Sciences (Shanghai, China). The HUVEC cell line was procured from Cobioer Biosciences Co., Ltd. (Nanjing, China). HuH-7, Hep3B, PLC/PRF/5, HepG2, SK-HEP-1 and MIHA cells were maintained in DMEM (C119955, Thermo, USA) comprising 10% fetal bovine serum (086-150, FBS, WISENT, Canada), 100 U/ml penicillin, and 100 μg/ml streptomycin (SV30010, HyClone, USA). SNU-387 and SNU-182 cells were kept in RPMI 1640 medium (C118755, Thermo, USA) with 10% FBS, 100 U/ml penicillin, and 100 μg/mL streptomycin. HUVECs were propagated in ECM medium (#1001, ScienCell, USA) comprising 1% EC growth supplement (ECGS), 5% FBS, 100 U/ml penicillin, and 100 μg/mL streptomycin. All cell lines were maintained at 37 °C in a humidified incubator with 5% CO_2 and verified to be free of mycoplasma contamination using the Myco-Blue Mycoplasma Detector (D101-01, Vazyme, China).

Plasmids

Short hairpin RNAs (shRNAs) targeting human ZCCHC4, TMEM97, SCD, HMGCR and Twist1 were inserted into the pLKO.1-Puro vector. A Scramble shRNA construct was employed as the negative control. The coding sequence (CDS) of FLAG-tagged ZCCHC4 was subcloned into the lentiviral pCDH-CMV-MCS-EF1-Puro vector. The CDS of human TMEM97, was likewise introduced into the pCDH-CMV-MCS-EF1-Blast vector. The CDS of LCN2 fused with a nuclear localization signal (NLS) peptide was subcloned into the pCDH-CMV-MCS-EF1-Puro vector. Furthermore, the promoter sequences of TMEM97, HMGCR, FASN, SCD and SQLE were individually subcloned into the pGL3-Basic vector to construct the corresponding reporter gene vectors, designated as pTMEM97, pHMGCR, pFASN, pSCD and pSQLE, in which firefly luciferase is expressed under the transcriptional control of the TMEM97, HMGCR, FASN, SCD and SQLE promoters, respectively.

Lentivirus production and infection

The designated plasmids underwent co-transfection with lentiviral packaging plasmids psPAX2 and pMD2.G into 293T cells through calcium phosphate methodology. After 48 h, the supernatants containing the virus were harvested. For lentiviral infection, the harvested lentiviral supernatants were added to the HCC cells when reaching 60–80% confluence, followed by 24 h incubation (37°C, 5% CO_2). Subsequently, Puromycin (Sigma, USA) served as the selection agent for obtaining stably transduced cells.

RNA isolation and quantitative real-time polymerase chain reaction (qRT-PCR)

Total RNA was extracted from samples using FreeZol Reagent (Vazyme, R711) according to the manufacturer's protocol. Isolated RNA was reverse transcribed to cDNA using HiScript III RT SuperMix kit (Vazyme, R323) following the manufacturer's recommended procedures. Then, qRT-PCR was performed using SYBR Green Master Mix (Yeasen, 11202ES) according to the manufacturer's instructions. The specific primers used for qRT-PCR were listed in Table S6.

Cell proliferation and colony formation assay

Cell proliferation assay: HCC cells were seeded at a density of 4–5 × 10^3 cells per well in 96-well plates. Following seeding, the confluence of the cells was continuously monitored for 100 hours using the Incucyte Live-Cell Analysis System (Essen Bioscience, USA). All experiments were performed with five biological replicates per trial to ensure statistical reliability.

For the colony formation assay, HCC cells were plated in 6-well plates at a density of 1000 cells per well and cultured in complete medium in a humidified incubator maintained at 37 °C with 5% CO_2. The culture medium was replenished at 3 – 4 days intervals, with cultivation maintained for 12–14 days. Afterward, when there were at least 50 cells for a single clone, the clones underwent fixation using 4%

paraformaldehyde for 30 min, succeeded by crystal violet staining (0.1%) for 2 h. The colonies were imaged and counted.

Flow cytometry analysis

HCC cells were dissociated into single-cell suspensions and counted. Subsequently, 1×10^6 cells were resuspended in 100 μl of medium containing 2% fetal bovine serum (FBS), and incubated with anti-CD13 antibodies (301704, Biolegend), anti-CD24 antibodies (555428,BD Biosciences) and anti-EPCAM antibodies (60136FI, Stem Cell) for 30 min on ice in the dark. After a single wash, the cells were resuspended in staining buffer containing DAPI (Sigma) for subsequent detection. Samples were then analyzed on a flow cytometer named CytoFLEX LX (Beckman), and the acquired data were processed using CytExpert software.

Tube formation assay

Human umbilical vein endothelial cells (HUVECs) were cultured in complete medium for 24 h and then synchronized by incubation in endothelial cell medium (ECM) containing 0.1% fetal bovine serum (FBS) for an additional 12 h to arrest the cells at the same cell cycle phase. Matrigel Basement Membrane Matrix (BD Biosciences) was diluted with serum-free ECM at a volume ratio of 1:1 to prepare the working solution, and a total of 20 μl of the diluted Matrigel working solution was added to each well of a 96-well plate, followed by incubation at 37 °C for 30 min to allow gelation. HUVECs were then seeded onto the solidified Matrigel layer at a density of 5×10^4 cells per well. The 96-well plate was subsequently incubated at 37 °C with 5% CO_2 for 4 h. Following incubation, cellular tube formation images were captured under an inverted light microscope (magnification, ×100 or ×200, as appropriate) at three randomly selected fields per well.

Nile red staining

To visualize lipid droplets, cultured cells seeded in 6-well plates were fixed with 4%

paraformaldehyde solution at room temperature, washed in 1xPBS prior to staining with 5 μg/ml Nile red solution (MCE, USA) for 15 min in the dark. After staining, cells were washed twice with 1× PBS and counterstained with DAPI. The images were visualized by immunofluorescence microscopy.

Supplemental Figure legends and Figures:

Figure S1. ZCCHC4 modulates the migratory and invasive capabilities of HCC cells, while exerting no influence on liver cancer stem cell characteristics or angiogenesis. (A, B) The expression level of ZCCHC4 in HCC cells and immortalized hepatocytes was determined by qRT-PCR (**A**) and Western blotting (**B**). **C.** Western blotting analysis of ZCCHC4 expression in HCC cells transduced with shRNAs against ZCCHC4 and ZCCHC4 overexpression vector. **D.** Wound healing assay was performed to evaluate the effect of ZCCHC4 on the migratory capacity of HCC cells. Scale bar, 100μm. **E**.Panoramic views of lung metastases illustrating the effects of ZCCHC4 knockdown or overexpression on lung metastasis of HCC cells. **F**. WB analysis was performed to evaluate the effect of ZCCHC4 on the protein expression of EMT marker Vimentin, MET marker E-Cadherin, and Claudin-1. **G.** IF staining was used to analyze the effect of ZCCHC4 on the expression of Vimentin, Claudin-1, and E-Cadherin in HCC cells. Scale bar, 100μm. **H.** IHC staining was performed to examine the expression of Vimentin, Claudin-1, and E-Cadherin in lung metastatic tumor tissues of mice with altered ZCCHC4 expression. Scale bars, 1,000 μm (upper panels) and 50 μm (lower panels). **I.** Tube formation assay was performed to evaluate the effect of ZCCHC4 on angiogenesis. Scale bar, 20μm. **J.** Flow cytometry (FACS) assay was performed to detect the effect of ZCCHC4 on the expression of liver cancer stem cell markers CD13, CD24, and EpCAM. Error bars represent mean ± SD. Statistical

significance was determined using one-way analysis of variance (ANOVA, A) and two-tailed unpaired Student's t-test (D, H–J).

Figure S2. The effect of ZCCHC4 on the transcriptional activity of *HMGCR*, *SQLE*, *FASN*, and *SCD*. A. IF staining of ZCCHC4 in HCC cells. Scale bar, 100μm. **B.** The GSEA of RNA-seq showed the genes regulated by ZCCHC4 were significantly related to EMT. **C**. Luciferase reporter gene assays were performed to investigate the effects of ZCCHC4 overexpression or knockdown on the transcriptional activities of HMGCR, SQLE, FASN and SCD. Error bars represent mean ± SD. Student's t-test was utilized to determine statistical significance (C).

Figure S3. ZCCHC4 promotes cholesterol and fatty acid accumulation in HCC cells**. A.** Levels of different types of cholesterol esters (ChE) in HCC cells transduced with shScramble or ZCCHC4 shRNA. **B.** Levels of different subtypes of phosphatidylcholine (PC) in HCC cells transduced with shScramble or ZCCHC4 shRNA. **C.** Levels of various types of triglycerides (TG) in HCC cells transduced with shScramble or ZCCHC4 shRNA. **D.** Nile red staining was performed to evaluate the effects of ZCCHC4 knockdown or overexpression on lipid droplet formation in HCC cells. Scale bar, 20μm. **E.** Panoramic views of lung metastases illustrating the effects of SCD and/or HMGCR knockdown of hepatocellular carcinoma in ZCCHC4-overexpressing cells. Error bars represent mean ± SD. Statistical significance was determined using two-tailed unpaired Student's t-test for panels A-C.

Figure S4. ZCCHC4 promotes HCC migration, invasion, and growth via transcriptionally activating TMEM97. A. Western blot analysis was performed to evaluate the effects of ZCCHC4 on the expression of TMEM97, GSK3β, phospho-GSK3β, and β-catenin. **B.** Western blotting assay was conducted to confirm the expression level of TMEM97 in ZCCHC4-knockdown HCC cells transduced with the

TMEM97 overexpression vector. **C.** Western blot assays were performed to examine the effects of TMEM97 knockdown and Wnt inhibitor treatment on ZCCHC4 expression in HCC cells, respectively. **D**. Panoramic views of lung metastases showing the effect of TMEM97 overexpression on lung metastasis of ZCCHC4-knockdown HCC cells. **E.** Cell proliferation assays were performed to examine the effects of ZCCHC4 knockdown on the proliferative capacity of HCC cells, as well as the rescue effect of TMEM97 overexpression on the proliferative capacity of ZCCHC4-knockdown HCC cells. **F.** Colony formation assays were performed to determine the effects of ZCCHC4 knockdown on the colony-forming ability of HCC cells, as well as the rescue effect of TMEM97 overexpression on the colony-forming ability of ZCCHC4-knockdown HCC cells. **G.** A subcutaneous xenograft mouse model was established to assess the effect of ZCCHC4 knockdown on HCC growth, as well as the restorative effect of TMEM97 overexpression on ZCCHC4 knockdown-induced alterations in HCC growth. Tumor volumes and weights were measured. **H.** Transwell assays were performed to investigate the effects of Wnt signaling inhibitor, fatty acid synthesis inhibitor, and/or cholesterol synthesis inhibitor on ZCCHC4 overexpression-induced alterations in the migratory and invasive capacities of HCC cells. **I.** Western blot assays were performed to detect the expression changes of TMEM97 in HCC cells with ZCCHC4 overexpression following SCD and/or HMGCR knockdown. Error bars represent mean ± SD. Statistical significance was determined using two-tailed unpaired Student's t-test for panels E-H.

Figure S5. Nuclear LCN2 overexpression reverses the effect of TMEM97 overexpression on the migration and invasion of HCC cells. A. CoIP assay was conducted to verify the interaction between TMEM97 and LCN2 in highly metastatic HCC cells. **B.** Transwell assays were performed to evaluate the effects of nuclear LCN2

co-overexpression on the migratory and invasive capacities of HCC cells with TMEM97 overexpression. Scale bar, 100μm. **C.** IF staining was performed to detect the subcellular localization of LCN2 in HCC cells following ZCCHC4 knockdown or overexpression. Scale bar, 100 μm. Error bars represent mean ± SD. Statistical significance was determined using a two-tailed unpaired Student's t-test (B).

Figure S1

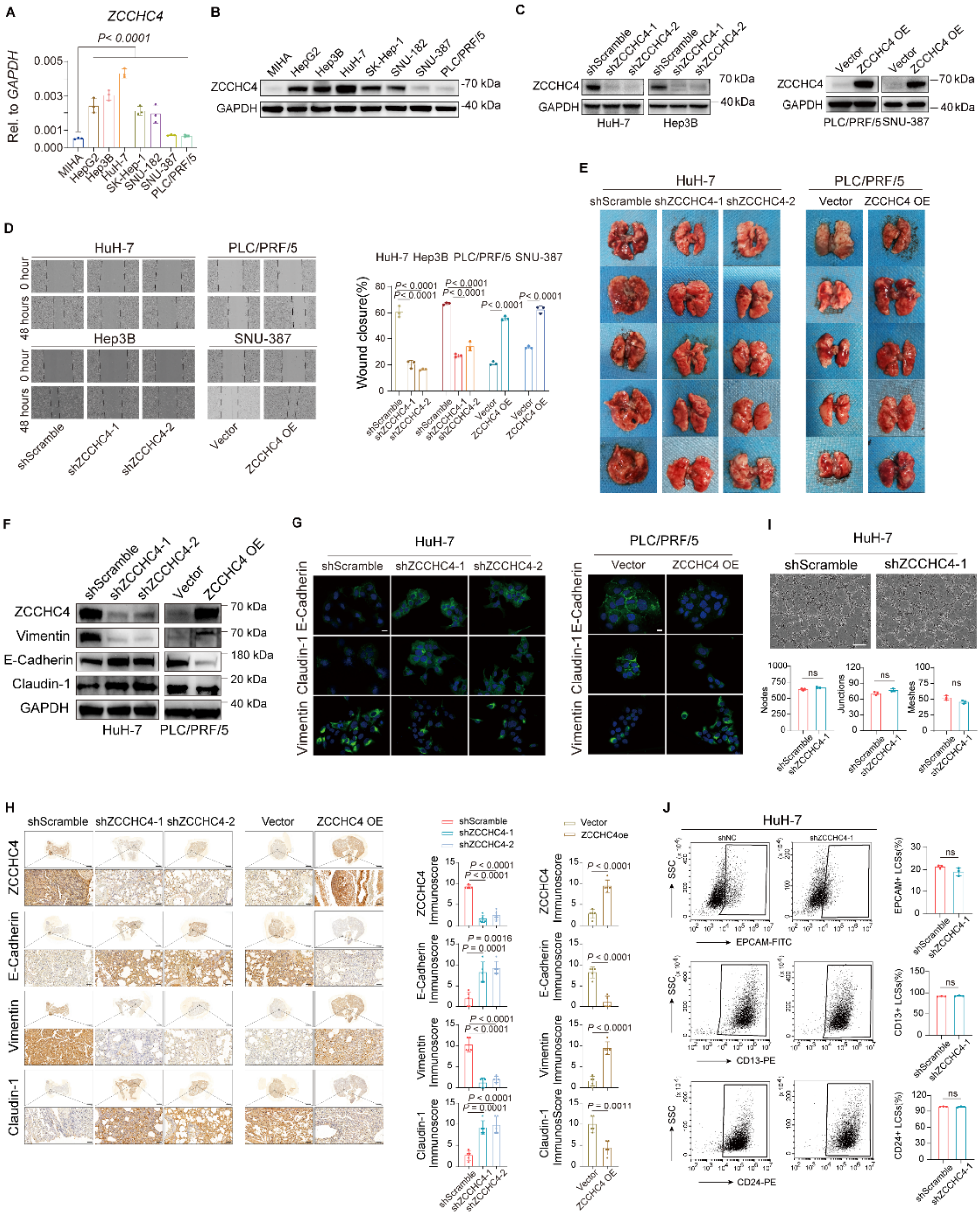

Fig S2

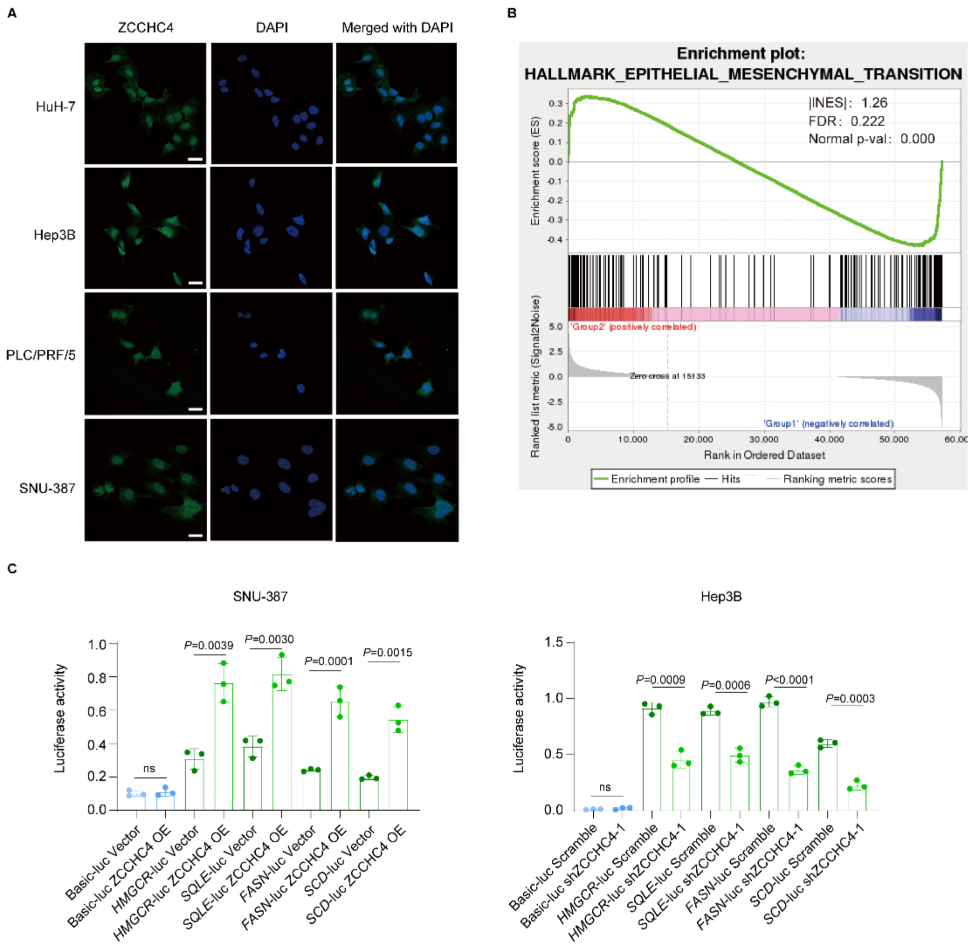
A
ZCCHC4
DAPI
Merged with DAPI
HuH-7
Hep3B
PLC/PRF/5
SNU-387
B
Enrichment plot:
HALLMARK_EPITHELIAL_MESENCHYMAL_TRANSITION
|NES|: 1.26
FDR: 0.222
Normal p-val: 0.000
Enrichment score (ES)
Ranked list metric (Signal2Noise)
Rank in Ordered Dataset
Enrichment profile
Hits
Ranking metric scores
C
SNU-387
Hep3B
Luciferase activity
P=0.0039
P=0.0030
P=0.0001
P=0.0015
P=0.0009
P=0.0006
P<0.0001
P=0.0003
ns
Basic-luc Vector
Basic-luc ZCCHC4 OE
HMGCR-luc Vector
HMGCR-luc ZCCHC4 OE
SQLE-luc Vector
SQLE-luc ZCCHC4 OE
FASN-luc Vector
FASN-luc ZCCHC4 OE
SCD-luc Vector
SCD-luc ZCCHC4 OE
Basic-luc Scramble
Basic-luc shZCCHC4-1
HMGCR-luc Scramble
HMGCR-luc shZCCHC4-1
SQLE-luc Scramble
SQLE-luc shZCCHC4-1
FASN-luc Scramble
FASN-luc shZCCHC4-1
SCD-luc Scramble
SCD-luc shZCCHC4-1

Fig S3

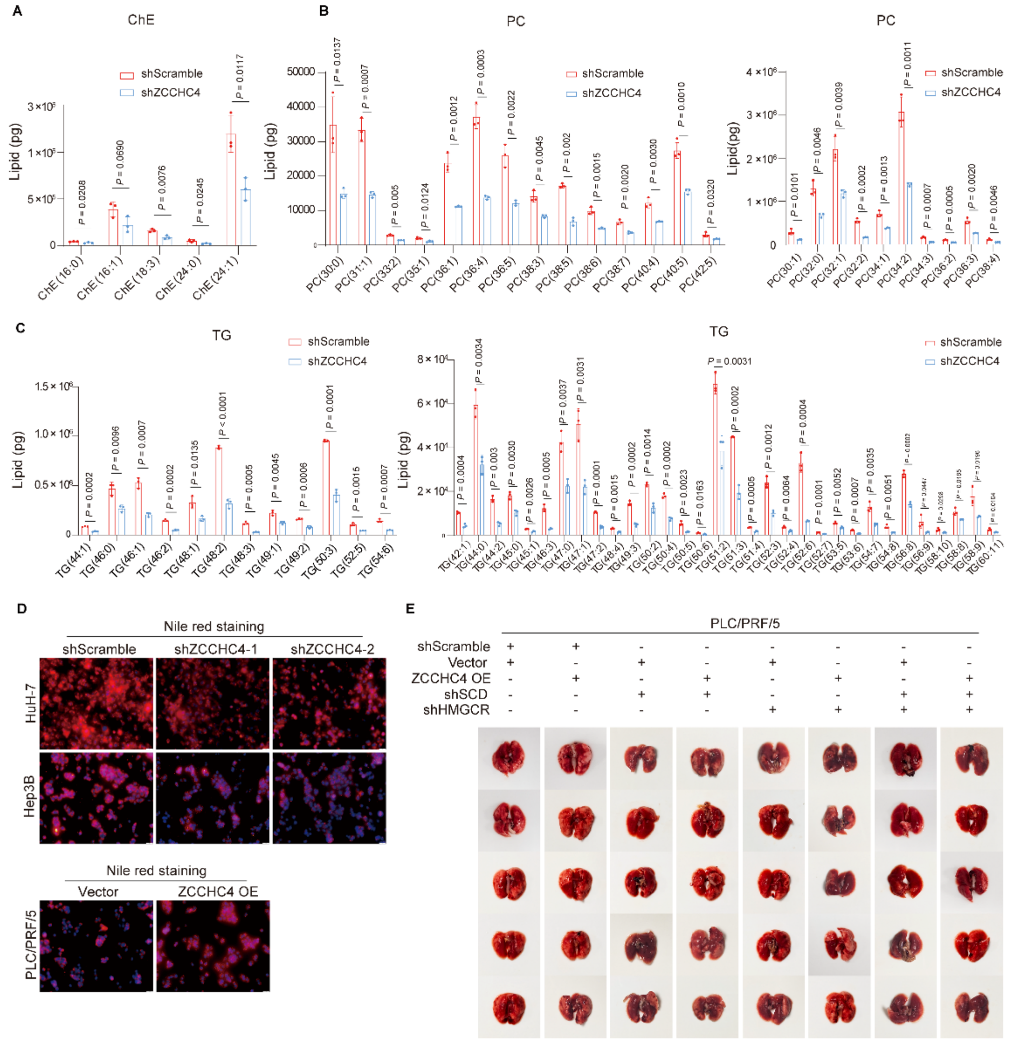
A
ChE
shScramble
shZCCHC4
Lipid (pg)
B
PC
Lipid(pg)
C
TG
D
Nile red staining
shScramble
shZCCHC4-1
shZCCHC4-2
HuH-7
Hep3B
Vector
ZCCHC4 OE
PLC/PRF/5
E
shScramble
Vector
ZCCHC4 OE
shSCD
shHMGCR

Figure S4

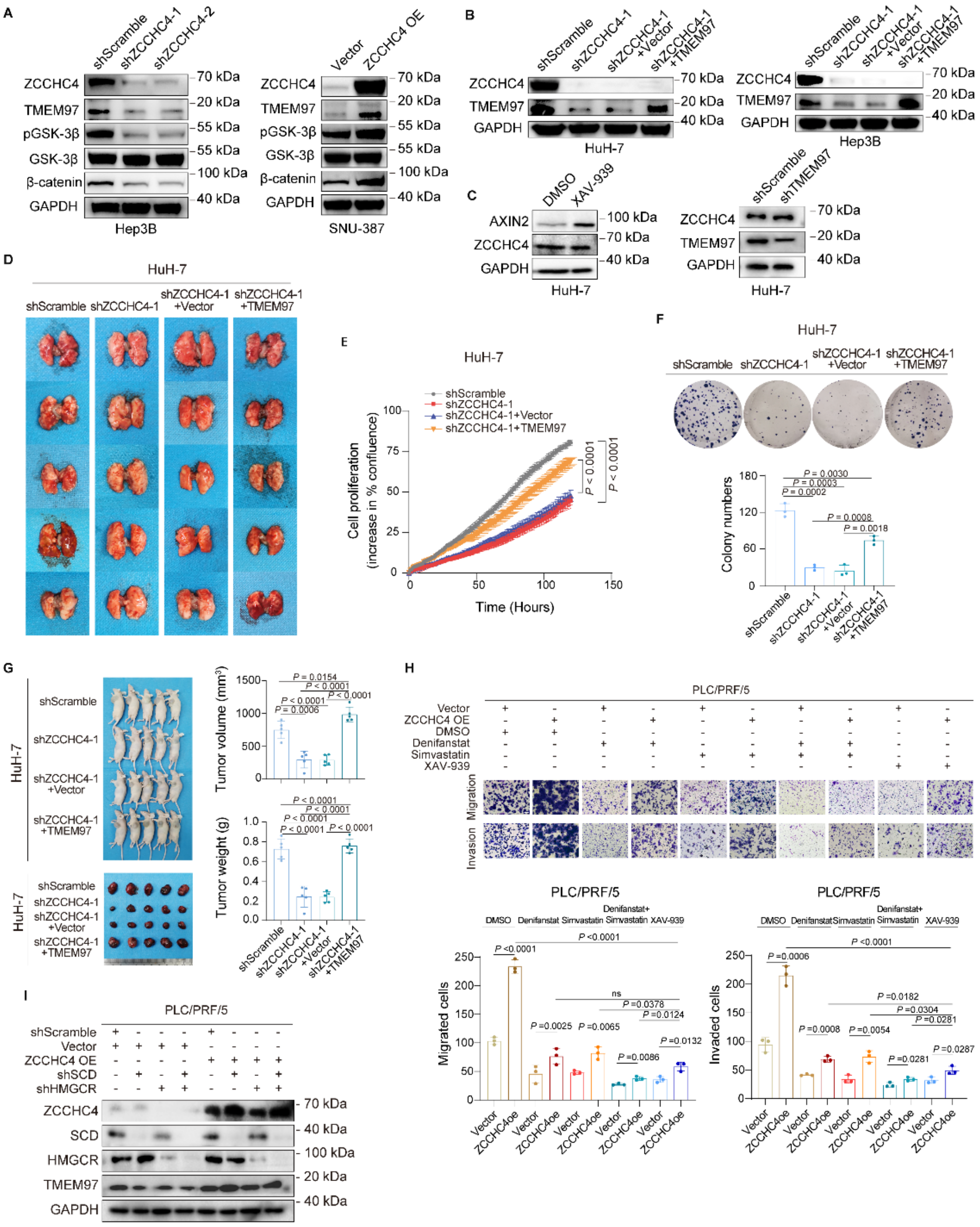
A
B
C
D
E
F
G
H
I
HuH-7
Hep3B
SNU-387
PLC/PRF/5
shScramble
ZCCHC4 OE
Cell proliferation (increase in % confluence)
Time (Hours)
Colony numbers
Tumor volume (mm³)
Tumor weight (g)
Migrated cells
Invaded cells
Migration
Invasion

Fig S5

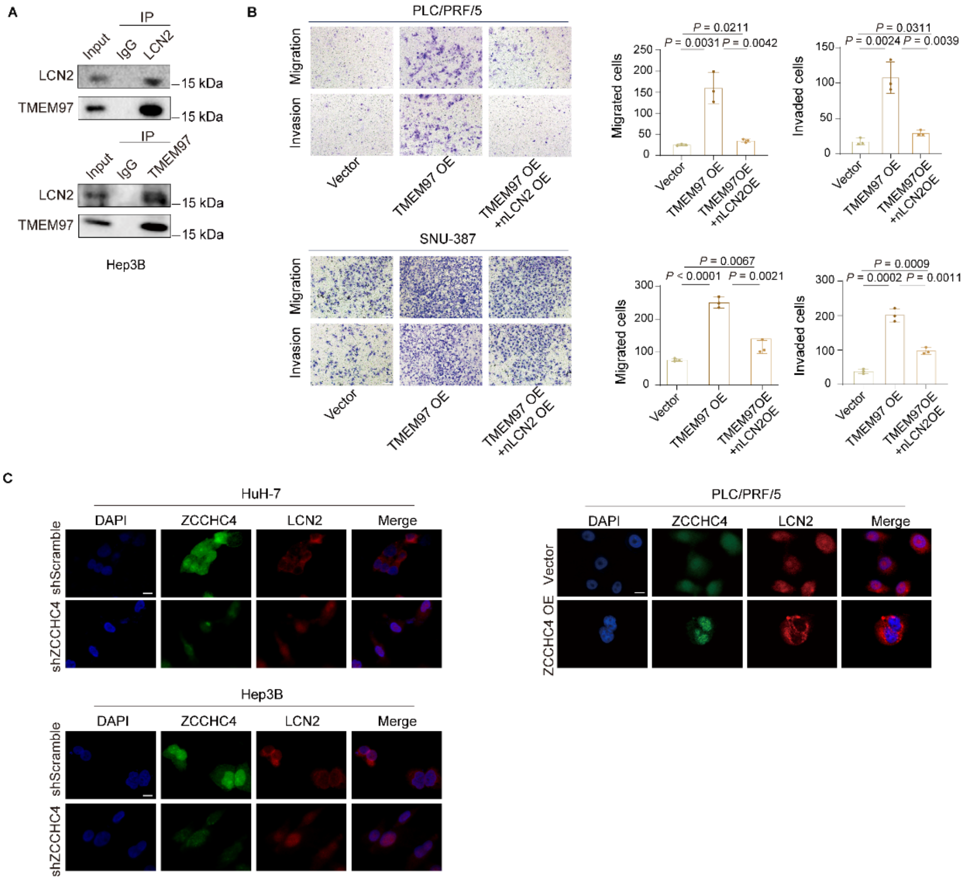
A
IP
Input
IgG
LCN2
LCN2
TMEM97
15 kDa
15 kDa
IP
Input
IgG
TMEM97
LCN2
TMEM97
15 kDa
15 kDa
Hep3B
B
PLC/PRF/5
Migration
Invasion
Vector
TMEM97 OE
TMEM97 OE +nLCN2 OE
P = 0.0211
P = 0.0031
P = 0.0042
Migrated cells
P = 0.0311
P = 0.0024
P = 0.0039
Invaded cells
TMEM97OE +nLCN2OE
SNU-387
P = 0.0067
P < 0.0001
P = 0.0021
P = 0.0009
P = 0.0002
P = 0.0011
C
HuH-7
DAPI
ZCCHC4
LCN2
Merge
shScramble
shZCCHC4
PLC/PRF/5
Vector
ZCCHC4 OE
Hep3B

Supplemental Table 1. Main clinical characteristics of HCC patients (95) studied in the project.

Characteristics	No. of Patients (%)
**Age (Years)**	
<60	71(74.7%)
≥60	24(25.3%)
**Gender**	
Male	80(84.2%)
Female	15(15.8%)
**Histological grade**	
G1	5(5.3%)
G2	70(73.7%)
G3	20(21.1%)
**Pathological Tumor (T) Status**	
T1-2	70(73.7%)
T3-4	25(26.3%)
**Pathological Node (N) Status**	
N0	89(93.7%)
N1	6(6.3%)
**Pathological metastasis (M) Status**	
M0	92(96.8%)
M1	3(3.2%)
**TNM stage**	
I-II	67(70.5%)
III-IV	28(29.5%)
**Encapsulation invasion**	
No	48(50.5%)
Yes	47(49.5%)
**Vascular invasion**	
No	46(48.4%)
Yes	49(51.6%)

Supplemental Table 2. Correlation between ZCCHC4 expression level and clinical parameters in 95 HCC patients.

Clinicopathologic parameter	Case	ZCCHC4 expression (%)		
		Low	High	P Value*
**Age (Years)**				
<60	71	16 (22.5)	55 (77.5)	0.125
≥60	24	2 (8.3)	22(91.7)	
**Gender**				
Male	80	16 (20)	64 (80)	0.545
Female	15	2 (13.3)	13 (86.7)	
**Histological grade**				
G1	5	2 (40)	3 (60)	0.119
G2	70	15 (21.4)	55 (78.6)	
G3	20	1 (5)	19 (95)	
**Pathological Tumor (T) Status**				
T1-2	70	18 (25.7)	52 (74.3)	**0.006**
T3-4	25	0 (0)	25 (100)	
**Pathological Node (N) Status**				
N0	89	16 (18)	73 (82)	0.353
N1	6	2 (33.3)	4 (66.7)	
**Pathological metastasis (M) Status**				
M0	92	18 (19.6)	74 (80.4)	0.395
M1	3	0 (0)	3 (100)	
**TNM stage**				
I-II	67	16 (23.9)	51 (76.1)	**0.016**
III-IV	28	2 (7.1)	26 (92.9)	
**Encapsulation invasion**				
No	48	15 (31.3)	33 (68.8)	**0.002**
Yes	47	3 (6.4)	44 (93.6)	
**Vascular invasion**				
No	48	15 (31.3)	33 (68.7)	**0.006**
Yes	47	3 (6.4)	44 (93.6)	

Supplemental Table 3. Univariate Cox regression Analysis for Disease-free Survival and Overall Survival in Patients with HCC.

Factors	Disease-free survival		Overall survival	
	**HR (95%CI)**	***p* value***	**HR (95%CI)**	***p* value***
Age, years (<60/≥60)	2.325（1.269-4.261）	**0.006**	2.516（1.361-4.649）	**0.003**
Gender (male/female)	0.615（0.243–1.558）	0.305	0.695（0.274–1.762）	0.444
Histological grade (1/2/3)	3.610（1.973-6.607）	**<0.001**	4.228（2.323-7.696）	**<0.001**
Tumor (T) status (1-2/3-4)	2.337（1.813-3.012）	**<0.001**	2.541（1.960-3.294）	**<0.001**
Nodal (N) status (0/1)	3.181（1.246-8.119）	**0.016**	2.248（0.864-5.849）	0.097
Metastasis (M) status (0/1)	9.155（2.584-32.431）	**0.001**	10.394（2.923-36.962）	**<0.001**
TNM stage （I-II/ III-IV）	2.198（1.706-2.833）	**<0.001**	2.083（1.631-2.660）	**<0.001**
Encapsulation invasion(yes/no)	2.153 (1.174-3.948)	**0.013**	2.273 (1.229-4.203)	**0.009**
Vascular invasion(yes/no)	11.102（4.891-26.581）	**<0.001**	7.904（3.655-17.090）	**<0.001**
ZCCHC4 expression (low/high)	3.786（2.024-7.080）	**<0.001**	3.701（1.978-6.927）	**<0.001**

Supplemental Table 4. Multivariate Cox regression analysis for disease-free survival and overall survival in patients with HCC.

Factors	Disease-free survival		Overall survival	
	HR (95%CI)	*p* value*	HR (95%CI)	*p* value*
Age, years (<60/≥60)	1.988（1.043-3.791）	**0.037**	2.577（1.335-4.974）	**0.005**
Histological grade (1/2/3)	1.862（0.903-3.837）	0.092	2.593（1.269-5.299）	**0.009**
Tumor (T) status (1-2/3-4)	1.753（1.044-2.944）	**0.034**	1.829（1.170-2.858）	**0.008**
Nodal (N) status (0/1)	3.048（0.674-13.793）	0.148		
Metastasis (M) status (0/1)	2.756（0.678-11.213）	0.157	10.394（2.923-36.962）	0.220
TNM stage (I-II/ III-IV)	0.724（0.353-1.485）	0.378	2.432（0.588-10.053）	**<0.001**
Encapsulation invasion(yes/no)	0.565 (0.276-1.155)	0.117	0.543 (0.256-1.151)	0.111
Vascular invasion(yes/no)	7.731（2.860-20.898）	**<0.001**	4.409（1.761-11.037）	**0.002**
ZCCHC4 expression (low/high)	2.693（1.321-5.489）	**0.006**	2.222（1.069-4.617）	**0.032**

Supplemental Table 5. The primer sequences for ChIP-qPCR.

F, Forward; R, reverse.

Name	Primer (5'-3')
TMEM97 F1 (F)	CAAGCCATCCTCCTGCCTCG
TMEM97 F1 (R)	AAGCTGACGCTGTGCACGGT
TMEM97 F2 (F)	TGTTGTAGTGGCGAACGGAG
TMEM97 F2 (R)	GCGTGGTATCCTGGTGTCCC
TMEM97 F3 (F)	CCAACCGACAGACTATGGGG
TMEM97 F3 (R)	TGGGGATGTGGCTGAGGAAG
TMEM97 F4 (F)	GCTGCGTGGAGTGGCTGCTG
TMEM97 F4 (R)	CCCCTCACCTCGACTGGGTA

Supplemental Table 6. The primer sequences for qRT-PCR.

F, Forward; R, Reverse.

Name	Primer (5'-3')
ZCCHC4(F)	CCCTCACGGACCCACTCTT
ZCCHC4(R)	GCAAGTCTAGCTCCTGACAAC
GAPDH(F)	GTGGACCTGACCTGCCGTCT
GAPDH(R)	GGAGGAGTGGGTGTCGCTGT
